# Circulating GDF11 exacerbates myocardial injury in mice and associates with increased infarct size in humans

**DOI:** 10.1093/cvr/cvad153

**Published:** 2023-09-23

**Authors:** Simon Kraler, Carolina Balbi, Daria Vdovenko, Tetiana Lapikova-Bryhinska, Giovanni G Camici, Luca Liberale, Nicole Bonetti, Candela Diaz Canestro, Fabienne Burger, Aline Roth, Federico Carbone, Giuseppe Vassalli, François Mach, Shalender Bhasin, Florian A Wenzl, Olivier Muller, Lorenz Räber, Christian M Matter, Fabrizio Montecucco, Thomas F Lüscher, Alexander Akhmedov

**Affiliations:** Center for Molecular Cardiology, University of Zurich, Wagistrasse 12, Zurich CH-8952, Switzerland; Center for Molecular Cardiology, University of Zurich, Wagistrasse 12, Zurich CH-8952, Switzerland; Laboratory of Cellular and Molecular Cardiology, Cardiocentro Ticino Institute, EOC, Lugano, Switzerland; Laboratories for Translational Research, EOC, Bellinzona, Switzerland; Center for Molecular Cardiology, University of Zurich, Wagistrasse 12, Zurich CH-8952, Switzerland; Center for Molecular Cardiology, University of Zurich, Wagistrasse 12, Zurich CH-8952, Switzerland; Center for Molecular Cardiology, University of Zurich, Wagistrasse 12, Zurich CH-8952, Switzerland; Department of Research and Education, University Hospital Zurich, Zurich, Switzerland; First Clinic of Internal Medicine, Department of Internal Medicine, University of Genoa, Genoa, Italy; IRCCS Ospedale Policlinico San Martino Genova—Italian Cardiovascular Network, Genoa, Italy; Center for Molecular Cardiology, University of Zurich, Wagistrasse 12, Zurich CH-8952, Switzerland; University Heart Center, Cardiology, University Hospital Zurich, Zurich, Switzerland; Center for Molecular Cardiology, University of Zurich, Wagistrasse 12, Zurich CH-8952, Switzerland; Division of Cardiology, Foundation for Medical Research, University of Geneva, Geneva, Switzerland; Division of Cardiology, Foundation for Medical Research, University of Geneva, Geneva, Switzerland; First Clinic of Internal Medicine, Department of Internal Medicine, University of Genoa, Genoa, Italy; IRCCS Ospedale Policlinico San Martino Genova—Italian Cardiovascular Network, Genoa, Italy; Center for Molecular Cardiology, University of Zurich, Wagistrasse 12, Zurich CH-8952, Switzerland; Laboratory of Cellular and Molecular Cardiology, Cardiocentro Ticino Institute, EOC, Lugano, Switzerland; Laboratories for Translational Research, EOC, Bellinzona, Switzerland; Division of Cardiology, Foundation for Medical Research, University of Geneva, Geneva, Switzerland; Research Program in Men's Health: Aging and Metabolism, Harvard Medical School, Brigham and Women’s Hospital, Boston, MA, USA; Center for Molecular Cardiology, University of Zurich, Wagistrasse 12, Zurich CH-8952, Switzerland; Department of Cardiology, University Hospital of Lausanne, University of Lausanne, Lausanne, Switzerland; Department of Cardiology, Inselspital Bern, Bern, Switzerland; Center for Molecular Cardiology, University of Zurich, Wagistrasse 12, Zurich CH-8952, Switzerland; University Heart Center, Cardiology, University Hospital Zurich, Zurich, Switzerland; First Clinic of Internal Medicine, Department of Internal Medicine, University of Genoa, Genoa, Italy; IRCCS Ospedale Policlinico San Martino Genova—Italian Cardiovascular Network, Genoa, Italy; Center for Molecular Cardiology, University of Zurich, Wagistrasse 12, Zurich CH-8952, Switzerland; Royal Brompton and Harefield Hospitals and Imperial College and Kings College, London, UK; Center for Molecular Cardiology, University of Zurich, Wagistrasse 12, Zurich CH-8952, Switzerland

**Keywords:** Acute myocardial infarction, Risk prediction, Proteomics, Ageing, GDF11, Biomarker discovery

## Abstract

**Aims:**

The heart rejuvenating effects of circulating growth differentiation factor 11 (GDF11), a transforming growth factor-β superfamily member that shares 90% homology with myostatin (MSTN), remains controversial. Here, we aimed to probe the role of GDF11 in acute myocardial infarction (MI), a frequent cause of heart failure and premature death during ageing.

**Methods and results:**

In contrast to endogenous *Mstn*, myocardial *Gdf11* declined during the course of ageing and was particularly reduced following ischaemia/reperfusion (I/R) injury, suggesting a therapeutic potential of GDF11 signalling in MI. Unexpectedly, boosting systemic Gdf11 by recombinant GDF11 delivery (0.1 mg/kg body weight over 30 days) prior to myocardial I/R augmented myocardial infarct size in C57BL/6 mice irrespective of their age, predominantly by accelerating pro-apoptotic signalling. While intrinsic cardioprotective signalling pathways remained unaffected by high circulating GDF11, targeted transcriptomics and immunomapping studies focusing on GDF11-associated downstream targets revealed attenuated Nkx2-5 expression confined to CD105-expressing cells, with pro-apoptotic activity, as assessed by caspase-3 levels, being particularly pronounced in adjacent cells, suggesting an indirect effect. By harnessing a highly specific and validated liquid chromatography-tandem mass spectrometry–based assay, we show that in prospectively recruited patients with MI circulating GDF11 but not MSTN levels incline with age. Moreover, GDF11 levels were particularly elevated in those at high risk for adverse outcomes following the acute event, with circulating GDF11 emerging as an independent predictor of myocardial infarct size, as estimated by standardized peak creatine kinase-MB levels.

**Conclusion:**

Our data challenge the initially reported heart rejuvenating effects of circulating GDF11 and suggest that high levels of systemic GDF11 exacerbate myocardial injury in mice and humans alike. Persistently high GDF11 levels during ageing may contribute to the age-dependent loss of cardioprotective mechanisms and thus poor outcomes of elderly patients following acute MI.

## Graphical Abstract

**Graphical Abstract:**
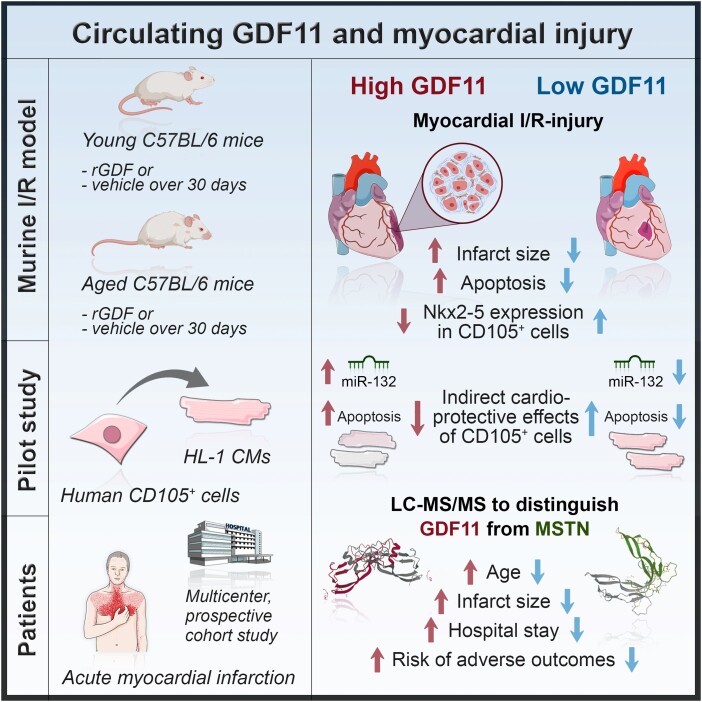

**Time of primary review: 25 days**

**Translational perspective**

Acute myocardial infarction (MI) is a frequent cause of heart failure and premature death in the elderly. Recent studies challenge the initial paradigm attributing growth differentiation factor 11 (GDF11) a heart rejuvenating function. Here, we show that, in stark contrast to the initial paradigm, systemic GDF11 replenishment exacerbates myocardial injury in a translational mouse model of MI. In line, circulating GDF11 increased as a function of age in patients with MI, with GDF11 emerging as an independent predictor of myocardial infarct size. Hence, high levels of GDF11 may contribute to the age-dependent loss of endogenous cardioprotective mechanisms, rendering elderly patients particularly susceptible for adverse outcomes following acute MI.

## 1. Introduction

Acute myocardial infarction (MI) spearheads mortality statistics around the globe. Despite the unprecedented gains over the past six decades,^1^ therapeutic progress has stalled more recently,^2,3^ specifically among the growing population of elderly and frail,^4,5^ a patient population at pronounced risk of extensive myocardial injury, fulminant heart failure, and premature death.^6,7^

Tissue damage due to MI is caused by both the ischaemic insult and subsequent reperfusion, a phenomenon referred to as ischaemia/reperfusion (I/R) injury. Endogenous cardioprotective mechanisms wane during cardiac ageing,^8^ rendering cardiomyocytes less tolerant to stress and increasingly susceptible for accelerated cell death upon I/R.^9^ The extent of myocardial tissue damage accrues from unregulated (necrosis) and regulated (apoptosis, necroptosis, and pyroptosis) modes of cell death, the latter being controlled by a variety of intra- and extracellular factors.^10,11^ In its early phase, apoptotic cell death is hallmarked by the maintenance of sarcolemmal integrity, does not elicit a pro-inflammatory response, and can be regulated by extracellular factors released by non-myocyte cells.^12–19^ The concept that the multilineage differentiation potential underpins the cardioprotective effects of CD105^+^ cells has long prevailed,^20,21^ but data of more recent studies by us^12,13^ and independent laboratories^14,16–19^ favour the involvement of paracrine factors released by CD105^+^ non-myocyte cells, including extracellular vesicles enriched in non-coding RNAs,^12^ therethrough contributing to cardiomyocytes’ resilience to ischaemic stress. The expression of genes responsive to transforming growth factor (TGF)-β signalling is confined to non-myocyte cells,^22^ with cardiac mesenchymal cells abundantly expressing CD105, a 180 kDa transmembrane glycoprotein that interacts with TGF-β signalling.^23–25^

Systemic levels of growth differentiation factor 11 (GDF11), a member of the TGF-β superfamily that shares 90% homology with myostatin (MSTN),^26^ were initially reported to decline with age,^27^ and systemic GDF11 replenishment by heterochronic parabiosis or recombinant GDF11 (rGDF11) delivery was postulated to have rejuvenating effects by reinstating regenerative capacity of aged muscle stem cells and hypertrophic cardiomyocytes.^27,28^ Yet, in light of insufficient target specificity, more recent studies challenged the initial paradigm, arguing that systemic levels of GDF11 do not decrease as a function of age,^29–33^ but may even incline during ageing,^29,32^ the latter being associated with frailty and adverse outcomes in elderly patients undergoing valve replacement therapy.^30^

The spatial expression patterns of GDF11 and MSTN differ markedly,^26,34^ notwithstanding MSTN and GDF11 governing similar signalling cascades, both acting on the SMAD2/3 pathway via binding to activin type II receptors and in turn inducing SMAD2/3 phosphorylation to repress genes required for cell differentiation.^35,36^ In mice bearing a *Mstn^Gdf^*^11^ knock-in allele, Gdf11 exhibits similar functional properties for the control of muscle mass; but the loss of murine *Gdf11* or *Mstn* results in distinct cardiac phenotypes,^36^ with *Mstn^−/−^* animals showing eccentric hypertrophy and enhanced β-adrenergic responsiveness.^37^ However, the role of *Gdf11* in the heart is less explored, partly because of the high perinatal lethality of *Gdf11^−/−^* mice which typically exhibit homeotic skeletal transformations and renal agenesis.^38,39^

In line with the originally reported inhibitory effect of GDF11 on human skeletal muscle–derived cell differentiation, Egerman *et al.*^29^ provided compelling evidence that the age-dependent increase in circulating GDF11 leads to inhibited rather than improved muscle regeneration, a finding confirmed in independent studies published thereafter.^40,41^ At the myocardial level, mild to no anti-hypertrophic effects by GDF11 were reported in young mice subjected to pressure overload–induced cardiomyopathy,^27,42^ and boosting systemic GDF11 levels by 4-week rGDF11 delivery failed to reverse age-related cardiac remodelling or to improve left ventricular function in independent studies,^43,44^ collectively questioning the notion of GDF11 as a heart rejuvenating factor.

Given the controversy of the effects of high circulating GDF11 on cardiac structure and function, we aimed to probe the effects of high systemic GDF11 on myocardial I/R injury by harnessing an array of methods, ranging from an established *in vivo* I/R model,^45^ omics-guided *in vitro* approaches^12,46,47^ to liquid chromatography-tandem mass spectrometry (LC-MS/MS)–based investigations of circulating GDF11 and MSTN levels in prospectively recruited patients with MI.^31,48–50^

## 2. Methods

Detailed methods descriptions are provided in the Supplementary material online.

### 2.1 Mouse model of I/R injury

Male C57BL/6 mice were obtained from Janvier Labs (Le Genest-Saint-Isle, France) and fed a standard chow (Kliba Nafag, Kaiseraugst, Switzerland). Mice were housed at 24°C under specific pathogen-free conditions with a 12 h light/dark cycle and *ad libitum* access to food and water. To determine the age dependency of myocardial *Gdf11* and *Mstn* expression, whole heart tissues of young (3–4 months), middle-aged (12–14 months), and aged (22–24 months) mice were freshly dissected and further processed as outlined in the Supplementary material online. Upon reviewing of these results (*n* = 5 per group), both young (3–4 months) and aged (22–24 months) mice were assigned to receive daily i.p. injections of either human rGDF11 [0.1 mg/kg body weight (BW); PeproTech, disulfide-linked homodimer comprising two 109 amino acid polypeptide chains; Supplementary material online, Key resources table] or volume-adjusted vehicle [0.1% bovine serum albumin (BSA) containing phosphate buffered saline (PBS)] over 30 days, a duration necessary to achieve significant elevations *in vivo*.^27^ At the end of the 30-day study period, mice were subjected to 30 min of ischaemia (I) followed by 15 min (protein isolation), 8 h (RNA isolation), or 24 h (morphological analyses, TUNEL assay) of reperfusion (R), before they were sacrificed, as reported previously.^51^ Briefly, following anaesthesia induction with 4% isoflurane, mice were intubated and mechanical ventilation with 100% oxygen was initiated using a tidal volume of 150 μL (120 breaths/min), during which anaesthesia was maintained by isoflurane inhalation. Depth of anaesthesia was continuously monitored, meanwhile a lateral blunt thoracotomy via the third intercostal space was performed to allow for the exposure of the pericardium. Following partial pericardiectomy, the left anterior descending (LAD) coronary artery was ligated using an 8–0 Prolene suture at the lower edge of the left atrium. To avoid laceration of the artery, a polyethylene tube was placed between the suture and the LAD. After a total of 30 min of successful LAD occlusion, the LAD was reopened to allow for myocardial reperfusion. Following full restoration of coronary blood flow, as confirmed by re-establishment of reddish-brown colour within the ischaemic area, the chest was closed, and anaesthesia was reduced to allow for spontaneous respiration. Analgesia was extended with subcutaneous injection of 0.05 mg/kg buprenorphine HCl every 4–6 h until sacrifice. After the indicated reperfusion periods, animals were then sacrificed and blood/cardiac tissues were further processed depending on the downstream experiments planned, as outlined in the Supplementary material online. All animal-related procedures were approved by the Cantonal Veterinary Authority, Switzerland, and conformed to the Directive 2010/63/EU of the European Parliament and of the Council of 22 September 2010 on the protection of animals used for scientific purposes.

### 2.2 Western blotting

Proteins from cardiac tissues or CD105^+^ cells were extracted using a protease inhibitor containing lysis buffer [50 mM Tris, pH 7.5; 1 mM ethylenediaminetetraacetic acid (EDTA), pH 8.0; 150 mM NaCl; 1 mM phenylmethylsulfonyl fluoride (PMSF), and 1 mM dithiothreitol (DTT)]. Protein extracts were then cleared by centrifugation, and total protein concentration was determined by a calorimetric method.^52^ Next, proteins were separated by gel electrophoresis on 8 or 10% sodium dodecyl sulphate (SDS)–polyacrylamide gel before being transferred to a polyvinylidene fluoride membrane (PVDF). After blocking with 5% skim milk or BSA, as appropriate, membranes were incubated with the indicated primary antibodies overnight at 4°C, followed by incubation with secondary horseradish peroxidase (HRP)–conjugated secondary antibodies (Birmingham, AL, USA) for 1 h at room temperature. Protein expression signals were detected using the Amersham Imager 600 (General Electric Healthcare Europe GmbH, Glattbrugg, Switzerland) and quantified by ImageJ 1.50 (National Institute of Health, Bethesda, MD, USA).

### 2.3 Real-time qPCR

Total RNA was isolated from whole tissues or cells with TRIzol (Invitrogen, Life Technologies Corporation, Zug, Switzerland) and reverse-transcribed into cDNA by using RT2 First Strand kit (QIAGEN, Hilden, Germany) according to the manufacturer’s instructions. Predesigned TaqMan Gene Expression Assays specific for *Mstn*, *Gdf11*, *Gata4*, and *Nkx2-5* were used throughout, with *Gapdh* serving as the reference gene. Respective controls (i.e. reverse transcription and genomic DNA contamination) were included in each run. Real-time qPCR run was performed on a Quant Studio 5 cycler (Thermo Fischer Scientific, Zug, Switzerland) using a standard amplification protocol. Target gene mRNA expression levels were normalized to *Gadph* using the comparative CT method.^53^

### 2.4 Serum assays

Frozen murine serum samples obtained 24 h after I/R were thawed on ice and immediately processed thereafter. Cardiac troponin I (cTnI) levels were measured by a high-sensitive enzyme-linked immunosorbent assay (ELISA; Life Diagnostics Inc.), with a lower limit of detection of 0.156 ng/mL. Serum levels of CXCL1 and CCL2 were measured by ELISA (R&D Systems, Minneapolis, MN, USA), following the manufacturer’s instructions, with the lower limit of detection being 15.6 and 7.8 pg/mL for CXCL1 and CCL2, respectively. Absorbance was measured on a plate reader (Infinite® 200 PRO, TECAN, Männedorf, ZH, Switzerland) set at 450 and 550 nm, respectively. Mean intra- and interassay coefficients of variation (CV) were below 6.0% for cTnI, CXCL1, and CCL2. Individual serum concentrations were calculated using a four-parameter logistic (4PL) curve fit.

### 2.5 Semi-quantitative immunohistochemistry

Freshly dissected hearts were fixed with 10% formaldehyde overnight at 4°C and embedded in paraffin blocks, after which tissues were sectioned at 3 μm intervals, immersed in BOND Epitope Retrieval Solution 2 at pH 9.0 and heat retrieved for up to 30 min at 100°C. To reduce background signals owing to hydrophobic and ionic interactions, tissue sections were blocked using a 0.4% casein-containing PBS solution. For the quantification of cell-specific Nkx2-5 protein expression, double-antibody immunohistochemistry (IHC) against CD105 and Nkx2-5 was performed using the Leica Bond-III Processing Module. In a separate batch of cardiac tissues, double-antibody IHC against CD105 and caspase-3 was performed. Tissue slides were incubated with primary antibodies for 60 min at room temperature, followed by incubation with alkaline phosphatase (AP)- and HRP-conjugated secondary antibodies, respectively. Tissue sections were developed using biotin-free BOND Polymer Refine Detection kits (see Supplementary material online, Key resources table). Semi-quantitative determination of protein expression was done using ImageJ Fiji (v.2.9.0), as previously described.^54–56^ In brief, each red–green–blue (RGB)-based double-antibody IHC image was split into three single-coloured images allowing for the separate quantification of Fast Red and 3,3'-diaminobenzidine (DAB) signals. Quantification of protein expression was done separately in CD105^+^ and CD105^−^ areas after adjusting the maximum threshold for each signal, the latter being constantly held at the same value for each marker across all images. To quantify the pro-apoptotic activity of cells adjacent to CD105^+^ cells, caspase-3 expression was determined in the pericellular region, defined as an area extending 9 × 9 pixels from CD105^+^ positively stained areas, with 1 pixel equalling roughly 1 µm. Results are presented as normalized values (calculated as mean intensity of the positively stained area divided by total image area) of heart tissues of rGDF11-treated mice relative to controls.

### 2.6 Clinical study

The SPUM-ACS study (ClinicalTrials.gov Identifier: NCT01000701) is an investigator-driven, multicentre prospective cohort study in Switzerland in which patients with suspected acute MI were recruited between December 2009 and 2017. Its study design and detailed inclusion and exclusion criteria have been reported previously.^48–50^ In all patients, blood sampling was done prior to coronary angiography. More details are provided in the Supplementary material online.

### 2.7 Proteomics for the detection of circulating GDF11 and MSTN

Circulating levels of GDF11 and MSTN in human or murine samples were measured by using a previously reported and externally validated LC-MS/MS method that employs sample denaturation (to ensure dissociation of MSTN and GDF-11 from their cognate binding proteins), reduction, alkylation, solid-phase extraction, and tryptic digestion, followed by separation and quantification using signature peptide-based multiple reaction monitoring and C-terminal [^13^C_6_^15^N_4_]-Arg peptides as internal standards.^31^ The intra- and interassay CV were 8.01–13.0% and 8.71–17.1% for GDF11, respectively, and 7.43–15.1% and 11.5–16.4% for MSTN, respectively. The lower limit of detection for each protein was 0.5 ng/mL. LC-MS/MS was done by fully blinded study personnel.

### 2.8 Statistical analysis

Data are presented as bar graphs with error bars [mean and standard error of the mean (SEM)] or as violin plots [median and interquartile range (IQR)], if skewed, with single data points superimposed. Between-group comparisons were performed by Student’s *t*-test, Mann–Whitney *U* test, one-way ANOVA, two-way ANOVA, Kruskal–Wallis *H* test, χ^2^ test, or Fisher’s exact test, as appropriate. If not stated otherwise, multiplicity-adjusted (Bonferroni) *P* values are reported throughout, with the threshold of α set at 0.05 (two-tailed). Data violating core assumptions of each test were either transformed or alternative (non-parametric) tests were used, as indicated in detail in the respective figure legends. More details on the hierarchical regression models are provided in the Supplementary material online. All analyses were performed using R version 4.2.1 (R Foundation for Statistical Computing, Vienna, Austria), SPSS version 28.0 (IBM, Armonk, NY, USA), or GraphPad Prism 8 (GraphPad Software, LLC, Massachusetts, USA).

## 3. Results

### 3.1 Myocardial *Gdf11* but not *Mstn* expression levels decrease with age and are blunted upon I/R injury in mice

**Figure 1:**
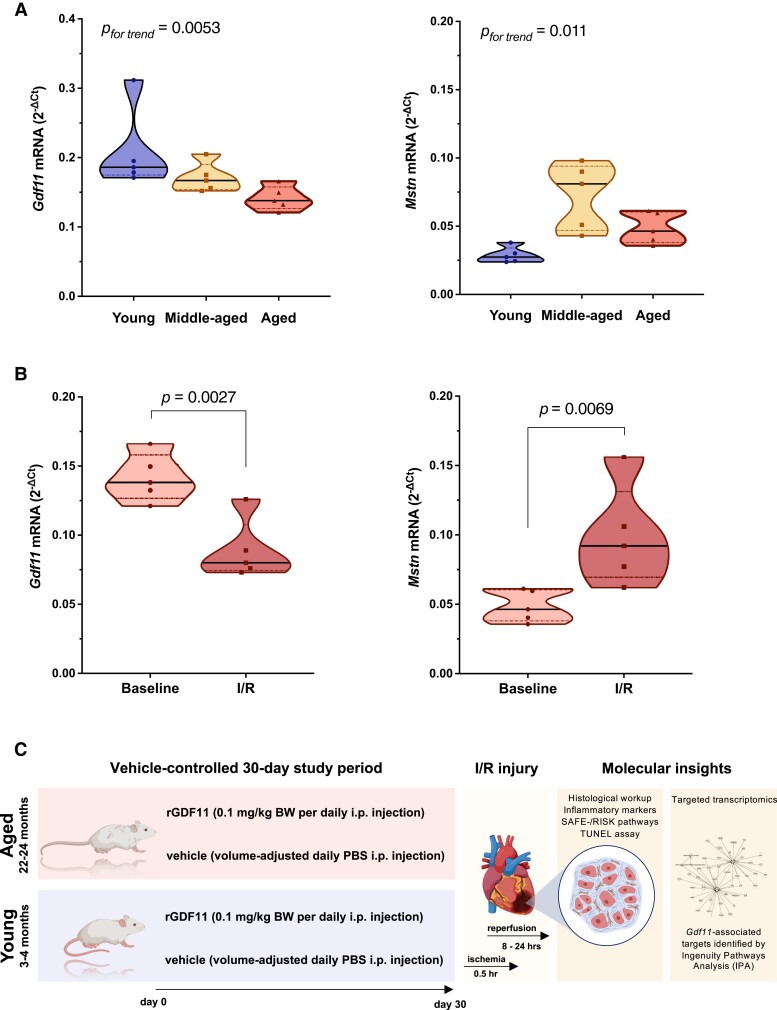
Age-mediated and myocardial I/R injury–mediated changes in Gdf11 and Mstn expression (top) and experimental study design (bottom). (*A*) Age-dependent changes in myocardial *Gdf11* and *Mstn* mRNA expression levels (*n* = 5/group). (*B*) Myocardial mRNA expression of *Gdf11* and *Mstn* of aged mice subjected to I/R injury or respective controls (*n* = 5/group). (*C*) Experimental scheme. Young (3–4 months) and aged (22–24 months) C57BL/6 mice were randomized to receiving rGDF11 (0.1 mg/kg BW daily i.p.) or vehicle (CTRL) over 30 days, as reported previously.^27^ Following I/R injury, morphological analyses and targeted transcriptomics were performed. *P* values were calculated by one-way ANOVA (*A*) or unpaired Student’s *t*-test (*B*). Data in *A* and *B* are presented as violin plots (median and IQR) with single data points superimposed.

We assessed myocardial *Gdf11* and *Mstn* expression in young (3–4 months), middle-aged (12–14 months), and aged (22–24 months) C57BL/6 mice. Notably, *Gdf11* expression declined as a function of age, whereas a linear trend towards increased *Mstn* expression levels was observed across age groups (*Figure 1A*). Although previous studies in fish and mice provided hints that *Gdf11* declines as the heart ages,^27,57,58^ the age dependency of myocardial *Gdf11* and *Mstn* expression has not been investigated systematically thus far.

Next, we sought to study myocardial expression levels of both *Gdf11* and *Mstn* in mice undergoing myocardial I/R injury and their respective controls. In line with previous reports in 8–10-week-old mice,^59,60^ a marked decline in *Gdf11* but an increase in *Mstn* expression was observed upon I/R (*Figure 1B*). Of note, Magga *et al.*^59^ have shown that these changes are accentuated early after I/R and are more prominent in the infarcted rather than the peri-infarcted area, suggesting that changes in endogenous *Gdf11* expression levels are predominantly induced by the ischaemic insult, and as such interfering with Gdf11 signalling may have therapeutic effects.

**Figure 2:**
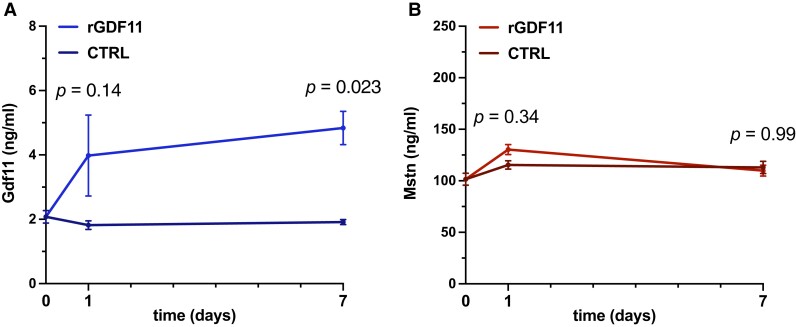
LC/LC-MS-based determination of circulating Gdf11 (left) and Mstn (right) upon rGDF11 delivery. (*A*) Daily injections of rGDF11 (0.1 mg/kg BW i.p./day; *n* = 11–16 mice/group) lead to a consistent increase in circulating Gdf11 after 7 days, as suggested by a previous report,^27^ and now, for the first time, confirmed by LC/LC-MS. (*B*) Importantly, circulating Mstn levels remain unaffected by rGDF11 supplementation. *P* values were calculated by two-way ANOVA. Data are shown as mean and SEM for each timepoint.

Based on the previously postulated rejuvenation effect of high systemic GDF11 on cardiac structure intertwined with the consistently observed I/R- and age-dependent decline in myocardial *Gdf11* across independent studies,^27,57–60^ we next sought to study the cardiac effects of boosting endogenous GDF11 by rGDF11 delivery prior to I/R injury in both young (3–4 months) and aged (22–24 months) animals. To that end, we performed a randomized, vehicle-controlled study in which C57BL/6 mice of both age groups were randomly assigned to receiving 0.1 mg/kg BW rGDF11 or vehicle daily over 30 days intraperitoneally (*Figure 1C*), a duration necessary to achieve significant elevations of circulating GDF11 *in vivo*, as confirmed by LC/LC-MS (*Figure 2A* and *B*).

### 3.2 Systemic GDF11 replenishment by rGDF11 increases experimental myocardial infarct size

**Figure 3:**
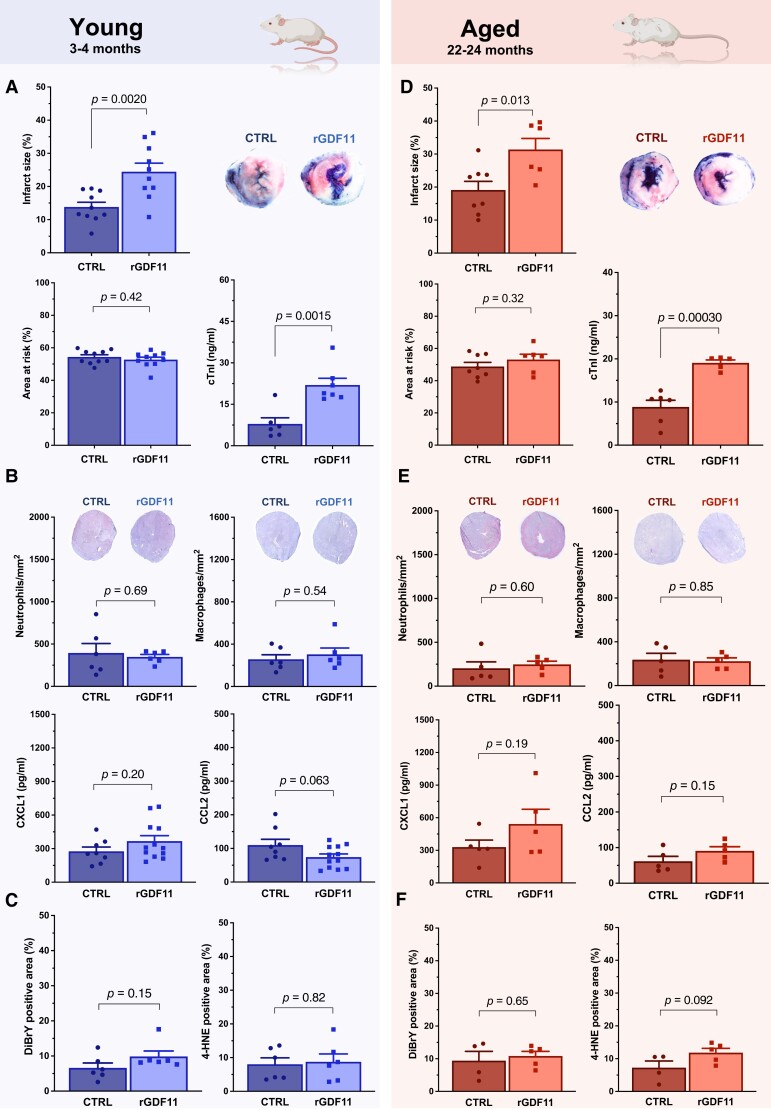
Histological and biochemical abnormalities of mice treated with rGDF11 or vehicle and subjected to myocardial I/R injury stratified by age. (*A*, *D*) Infarct size (I) and area at risk (AAR) relative to ventricle surface (calculated as I/V and AAR/V, respectively). cTnI denotes cardiac troponin I. Representative images of triphenyltetrazolium chloride-stained middle heart sections of vehicle- vs. rGDF11-treated mice are shown. (*B*, *E*) Quantification of neutrophil and macrophage infiltration together with representative histology (top) and CXCL1 and CCL2 serum levels (bottom) following acute myocardial I/R injury. (*C*, *F*) DiBrY- and 4-HNE-positive areas in tissue sections of infarcted hearts of both groups. *P* values were calculated by unpaired Student’s *t*-test (*A–F*). Data are presented as bar graphs and error bars (mean and SEM) with single data points superimposed.

Unexpectedly, young (3–4 months) mice receiving 30-day rGDF11 supplementation showed larger infarcts and higher cTnI levels following myocardial I/R as compared to vehicle-treated controls (CTRL), despite similar areas at risk (AAR; *Figure 3A*). Immunostaining pinpointed similar numbers of Ly-6G- and CD68-positive cells (i.e. neutrophils and macrophages, respectively) in cardiac tissues of both rGDF11 and CTRL mice, with no difference in blood-borne chemokine C-X-C motif ligand 1 (CXCL1) or monocyte chemoattractant protein 1 (CCL2) levels, key players of the acute inflammatory response evoked by I/R (*Figure 3B*).^10,61^ Similarly, surrogates of oxidative stress such as 4-hydroxy-2-nonenal (4-HNE) and 3,5-dibromotyrosine (DiBrY)-positive areas remained unchanged between rGDF11 and CTRL animals (*Figure 3C*).

In line with the results obtained in 3–4-month-old animals, aged (22–24 months) mice subjected to systemic GDF11 replenishment showed a similar increase in infarct size, as confirmed by histology and biochemical analyses (*Figure 3D*). Accordingly, the abundancy of neutrophils and macrophages did not differ between rGDF11 and CTRL animals, underscored by unchanged serum levels of both CXCL1 and CCL2 (*Figure 3E*). Moreover, the extent of DiBrY- and 4-HNE-positive areas remained unaltered between rGDF11- and vehicle-treated animals (*Figure 3F*), implying that the mechanisms involved act independently of acute inflammation and oxidative stress. This is of particular relevance, as enhanced reactive oxygen species (ROS) formation, as it occurs with I/R,^62,63^ represents a key feature of unregulated cell death.^10^ In contrast, both necroptosis and pyroptosis are characterized by necrosome and inflammasome activation, thus hallmarked by the activation of pro-inflammatory pathways via the release of inflammatory mediators, including immune cell–derived chemokines.^64,65^

### 3.3 High extracellular GDF11 triggers myocardial injury through accelerated cardiomyocyte apoptosis

**Figure 4:**
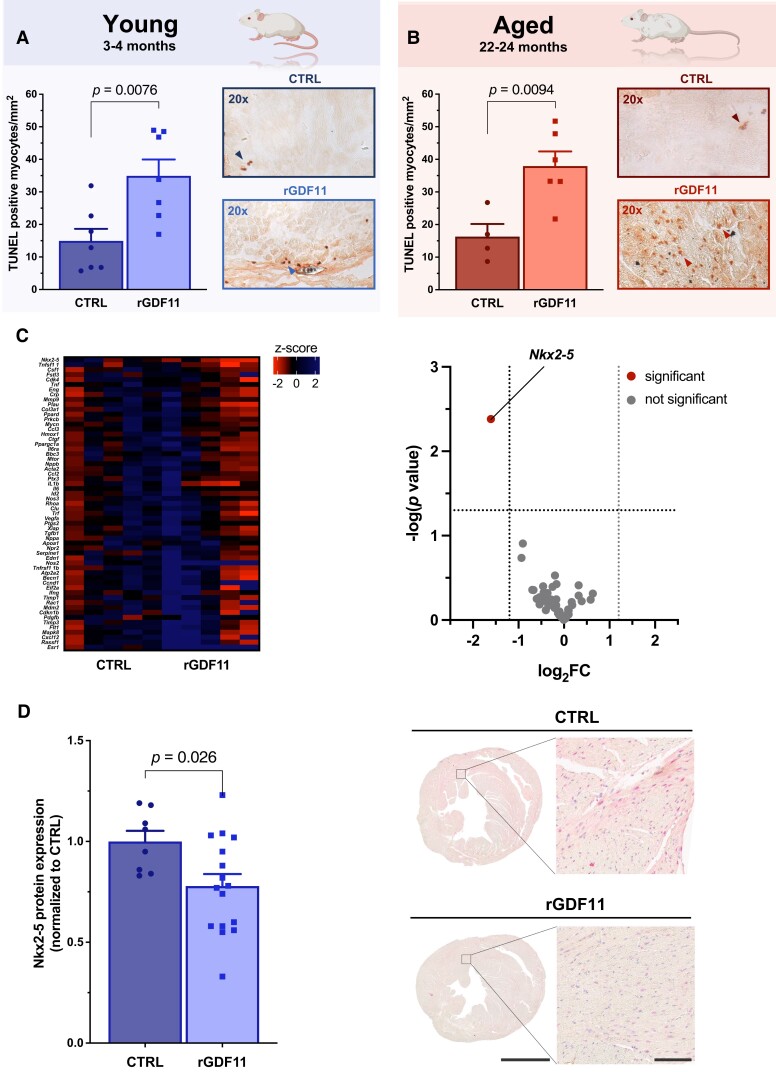
Systemic GDF11 restoration accelerates cardiomyocyte apoptosis with IPA-guided transcriptomics and immunostaining pointing towards a possible indirect effect mediated by non-myocyte cells. (*A*, *B*) Number of TUNEL-positive cardiomyocytes in young (3–4 months) and aged (22–24 months) mice. Representative images (×20) are shown on the right of each panel. (*C*) Left: heatmap represents *Z*-scored cardiac expression of IPA-identified genes in mice subjected to I/R injury and pre-treated with vehicle (CTRL) or rGDF11 (0.1 mg/kg BW i.p./day) over 30 days; right: volcano plot showing gene expression of IPA-identified candidates between both groups. (*D*) Semi-quantitative IHC shows attenuated Nkx2-5 protein expression in rGDF11 pre-treated mice. Scale bars: 2 mm (left panel), 100 μm (right panel). *P* values were calculated by (*A*, *B*) unpaired Student’s *t*-test or (*C*) multiple unpaired two-sample *t*-tests with Welch’s correction. Data (*A*, *B*, *D*) are presented as bar graphs and error bars (mean and SEM) with single data points superimposed.

High circulating GDF11 increased myocardial infarct size by 77 and 64% in young [24.45 vs. 13.81 I/V (infarct area/ventricle surface); *P* = 0.0020] and aged mice (31.36 vs. 19.11 I/V; *P* = 0.013), respectively; meanwhile, systemic GDF11 replenishment increased the abundancy of TUNEL-positive cardiomyocytes by 134% (34.94 vs. 14.94 TUNEL-positive myocytes/mm^2^; *P* = 0.0076; *Figure 4A*) and 133% (37.93 vs. 16.28 TUNEL-positive myocytes/mm^2^; *P* = 0.0094; *Figure 4B*), respectively, cumulatively pointing towards enhanced pro-apoptotic signalling as the main determinant of accelerated cell death in rGDF11-treated mice. Of note, a previous study found that exogeneous GDF11 upregulates the pro-apoptotic and mitophagy-associated gene *Bnip3*, coinciding with a pronounced loss of cardiac function over a 14-day period.^66^ Albeit duration and type of GDF11 supplementation are difficult to compare, these data suggest that exogeneous GDF11 delivery may prime cardiomyocytes to be more subject to apoptosis at basal states, which becomes morphologically evident if acute myocardial stress is induced by I/R injury.

The reperfusion injury salvage kinase (RISK) and the survival activating factor enhancement (SAFE) pathways are complex signalling cascades regulating cardiomyocyte survival in the setting of I/R involving cytosolic mediators.^10^ Notably, protein expression and/or phosphorylation status of important hub molecules,^10^ including Akt, p44/p42-MAPK, and Stat1, remained unchanged between groups (see Supplementary material online, *Figure S1*), implying distinct causative mechanisms. Given the variety of intra- and extracellular effectors potentially triggering accelerated pro-apoptotic signalling in the presence of high GDF11, ingenuity pathway analysis (IPA)-guided transcriptomics using total RNA extracts of hearts dissected from animals undergoing I/R were performed. Of all candidate genes tested, only the expression of the homeobox-containing transcription factor *Nkx2-5* differed significantly between groups (*Figure 4C*; see Supplementary material online, *Table S1*), which we confirmed quantitatively by both qPCR and Western blotting in an independent cohort of mice (see Supplementary material online, *Figure S2*). *Nkx2-5* is essential for looping morphogenesis of the heart tube and cardiac growth during embryogenesis,^67,68^ though myocardial *Nkx2-5* remains ubiquitously expressed during the postnatal stage. CD105^+^ cells were reported to show high *Nkx2-5* mRNA expression relative to cardiac fibroblasts,^69^ but total Nkx2-5 protein expression was interestingly not limited to one particular cell type homing the adult heart (*Figure 4D*).

Importantly, however, differential Nkx2-5 protein expression was only present in CD105^+^ but not in CD105^−^ heart regions, collectively suggesting that GDF11-mediated transcriptional changes predominantly occur in CD105^+^ cells (see Supplementary material online, *Figure S3*). Meanwhile, systemic GDF11 replenishment induced accentuated caspase-3 expression in cell regions adjacent to CD105^+^ cells, while this effect was not observed in pericellular regions of CD105^−^ cells (see Supplementary material online, *Figure S4*). With intrinsic cardioprotective signalling cascades remaining unaffected and pro-apoptotic pathways being selectively activated in cardiac cells adjacent to resident CD105^+^ cells, these hypothesis-generating data suggest that accelerated cell death by high circulating GDF11 may involve indirect (e.g. paracrine-mediated) mechanisms.^12–14,16–19^ Given the unavailability of suitable mouse models to provide direct experimental evidence on the role of resident CD105^+^ cells in GDF11-mediated I/R injury, an *ex vivo* pilot study (involving cardiac-specific CD105^+^ cells and HL-1 cardiomyocytes) was conducted whose results are provided in the Supplementary Material (see Supplementary material online, *Figures S5 and S6*).

### 3.4 Circulating GDF11 but not MSTN inclines with age and associates with increased infarct size in patients with acute MI

To assess the translational potential of our experimental findings, we next determined circulating GDF11 and MSTN protein levels in prospectively recruited patients with acute MI (*n* = 100; SPUM-ACS; ClinicalTrials.gov Identifier: NCT01000701) (*Table 1*; see Supplementary material online, *Figure S7* and *Table S2*). To accurately distinguish GDF11 from its close homolog MSTN, a highly specific and externally validated LC-MS/MS assay was used.^31^ Blood sampling was done prior to coronary intervention, after a median of 291 min following pain onset (*Table 2*). Of note, patients in the upper GDF11 tertile were older (*P* = 0.011), at higher risk of death at 6 months, as assessed by GRACE 2.0 (*P* = 0.036), and were hospitalized for longer (*P* = 0.011), implying that high circulating GDF11 levels associate with worse outcomes and high susceptibility for adverse events post-MI. Nonetheless, coronary lesion characteristics, major determinants of infarct size,^70,71^ were not different across GDF11 tertiles (*Tables 1 and 2*). Importantly, the associations noted above were absent if patients were stratified according to MSTN tertiles (see Supplementary material online, *Tables S3 and S4*).

**Table 1:** Baseline characteristics of SPUM-ACS study participants with acute myocardial infarction stratified by GDF11 tertiles

	All patients	GDF11 tertile 1	GDF11 tertile 2	GDF11 tertile 3	*P* value
	*n* = 100	<2.79 ng/mL (*n* = 33)	2.79–3.33 ng/mL (*n* = 34)	>3.33 ng/mL (*n* = 33)	
Clinical features at presentation					
Age (years)	66.4 (56.2–77.2)	64.7 (52.5–76.9)	61.9 (51.2–69.6)	69.9 (64.0–79.9)	0.011^a^
Female	23/100 (23.0)	9/33 (27.3)	5/34 (14.7)	9/33 (27.3)	0.368^c^
Heart rate (b.p.m.)	78 (70–91)	78 (70–91)	79 (69–94)	78 (70–91)	0.679^b^
Systolic blood pressure (mmHg)	122 (106–140)	120 (114–137)	118 (101–139)	129 (104–150)	0.438^b^
eGFR (mL/min/1.73 m^2^)	79.3 (64.4–94.2)	82.1 (64.3–93.9)	79.4 (70.9–99.0)	77.0 (47.8–90.6)	0.384^b^
Hs-cTnT >99th percentile	95/100 (95.0)	32/33 (97.0)	32/34 (94.1)	31/33 (93.9)	0.803^c^
LVEF (%)	48.0 (40.0–60.0)	50.0 (35.0–65.0)	50 (45.0–60.0)	45.0 (40.0–50.0)	0.107^b^
Killip class					
I	81/98 (82.7)	27/33 (81.8)	27/34 (79.4)	27/31 (87.1)	0.905^b^
II	10/98 (10.2)	4/33 (12.1)	4/34 (11.8)	2/31 (6.5)	
III	7/98 (7.1)	2/33 (6.1)	3/34 (8.8)	2/31 (6.5)	
IV	1/98 (1.0)	0/33 (0.0)	0/34 (0.0)	1/31 (3.2)	
Cardiometabolic risk factors					
BMI (kg/m^2^)	26.7 (24.2–29.1)	25.5 (22.8–27.7)	26.5 (24.2–28.7)	27.2 (25.2–30.7)	0.271^a^
BSA_c_ (m^2^)	1.9 (1.8–2.0)	1.8 (1.7–2.0)	1.9 (1.8–2.0)	1.9 (1.8–2.0)	0.230^a^
Current smoker	35/100 (35.0)	13/33 (39.4)	13/34 (38.2)	9/33 (27.3)	0.814^c^
Total cholesterol (mmol/L)	4.6 (3.9–5.4)	4.5 (3.9–5.5)	4.7 (4.4–5.2)	4.5 (3.7–5.6)	0.900^b^
LDL-C (mmol/L)	2.9 (2.3–3.5)	3.0 (2.1–3.9)	3.0 (2.7–3.3)	2.8 (2.4–3.4)	0.701^a^
HDL-C (mmol/L)	1.1 (1.0–1.4)	1.2 (1.0–1.4)	1.1 (0.9–1.3)	1.2 (1.0–1.4)	0.320^b^
Triglycerides (mmol/L)	1.0 (0.7–1.4)	0.9 (0.7–1.4)	1.0 (0.8–1.5)	1.0 (0.7–1.3)	0.843^b^
Past medical history					
H_x_ of dyslipidaemia	57/100 (57.0)	22/33 (66.7)	17/34 (50.0)	18/33 (54.5)	0.364^c^
H_x_ of hypertension	53/100 (53.0)	16/33 (48.5)	18/34 (52.9)	19/33 (57.6)	0.688^c^
Previous PCI	12/100 (12.0)	2/33 (6.1)	4/34 (11.8)	6/33 (18.2)	0.324^c^
Clinical chemistry and haematology					
hs-CRP (mg/L)	2.5 (1.2–9.4)	1.9 (0.8–6.3)	2.9 (1.1–10.0)	2.7 (1.7–9.3)	0.440^b^
NT-proBNP (ng/L)	331.0 (115.8–1349.5)	237.0 (126.0–1500.0)	444.0 (113.3–1147.5)	339.0 (123.0–1387.0)	0.880^b^
hs-cTnT (ng/L)	246.0 (71.0–705.0)	340.0 (65.0–622.0)	323.0 (113.5–886.5)	203.0 (55.0–587.0)	0.533^b^
Hb (g/dL)	13.5 (12.8–14.3)	13.7 (12.8–14.3)	13.5 (12.8–14.3)	13.4 (12.6–14.2)	0.300^b^
Medication at presentation					
Aspirin	23/52 (44.2)	5/17 (29.4)	11/19 (57.9)	7/16 (43.8)	0.228^c^
ACEI/ARB	27/52 (51.9)	12/17 (70.6)	10/19 (52.6)	5/16 (31.3)	0.077^c^
Beta-blocker	26/52 (50.0)	10/17 (58.8)	11/19 (57.9)	5/16 (31.3)	0.197^c^
P2Y_12_ receptor inhibitor	5/52 (9.6)	2/17 (11.8)	2/19 (10.5)	1/16 (6.3)	0.853^c^
Statin	19/52 (36.5)	3/17 (17.6)	11/19 (57.9)	5/16 (31.3)	0.038^c^

Data are *n*/*N* (%) or median (IQR).

ACEI, angiotensin-converting enzyme inhibitors; ARB, angiotensin receptor blockers; BMI, body mass index; BSA, estimated (by Dubois and Dubois) body surface area; CAD, coronary artery disease; CABG, coronary artery bypass grafting; CRP, C-reactive protein; eGFR, estimated [by Chronic Kidney Disease Epidemiology Collaboration (CKD-EPI)] glomerular filtration rate; GDF11, growth differentiation factor 11; GRACE, Global Registry of Acute Coronary Events; Hb, haemoglobin; LVEF, left ventricular ejection fraction; MSTN, myostatin; NT-proBNP, N-terminal-pro hormone BNP; PCI, percutaneous coronary intervention; URL, upper reference limit.

^a^One-way ANOVA.

^b^Kruskal–Wallis *H* test.

^c^χ^2^ test or Fisher’s exact test.

**Table 2:** Risk of adverse events, lesion characteristics, discharge medications, and MACE rates of all patients with MI according to GDF11 tertiles

	All patients	GDF11 tertile 1	GDF11 tertile 2	GDF11 tertile 3	*P* value
	*n* = 100	<2.79 ng/mL (*n* = 33)	2.79–3.33 ng/mL (*n* = 34)	>3.33 ng/mL (*n* = 33)	
GRACE 2.0 score (%)					
Death in hospital	3.2 (1.5–5.1)	2.6 (1.3–5.0)	3.1 (1.3–4.6)	3.8 (2.3–7.7)	0.128^a^
Death at 6 months	9.0 (4.0–15.0)	8.0 (4.0–15.0)	7.0 (4.0–12.8)	11.0 (6.0–20.0)	0.036^a^
Death at 1 year	6.3 (3.7–12.7)	5.7 (2.8–11.7)	6.0 (3.7–8.6)	8.1 (4.8–16.0)	0.061^a^
Management delay					
Onset-to-PCI (min)	291.0 (177.0–517.3)	201.0 (145.5–664.0)	305.0 (224.5–460.5)	275.0 (191.3–490.3)	0.805^a^
Lesion characteristics					
N_o_ of lesions^d^	1.0 (1.0–2.0)	1.0 (1.0–2.0)	1.0 (1.0–2.0)	1.0 (1.0–2.0)	0.814^a^
N_o_ of lesions stented^d^	1.0 (1.0–2.0)	1.0 (1.0–1.0)	1.0 (1.0–2.0)	1.0 (1.0–1.3)	0.806^a^
LAD occlusion	25/100 (25.0)	11/33 (33.3)	6/34 (17.6)	8/33 (24.2)	0.331^b^
Proximal lesion	9/100 (9.0)	5/33 (15.2)	1/34 (2.9)	3/33 (9.1)	0.186^b^
Total occlusion by main lesion^e^	51/90 (56.7)	15/30 (50.0)	17/32 (53.1)	19/28 (67.9)	0.206^b^
Main lesion morphology					
AHA/ACC type A	8/90 (8.9)	3/30 (10.0)	2/32 (6.3)	3/28 (10.7)	0.928^a^
AHA/ACC type B	63/90 (70.0)	19/30 (63.3)	24/32 (75.0)	20/28 (71.4)	
AHA/ACC type C	19/90 (21.1)	8/30 (26.7)	6/32 (18.8)	5/28 (17.9)	
Duration of hospital stay (days)	2.0 (1.0–2.25)	1.0 (1.0–2.0)	2.0 (1.0–3.0)	2.0 (1.0–2.0)	0.011^a^
Discharge destination					
Rehabilitation/other hospital	72/99 (72.7)	26/33 (78.8)	19/33 (57.6)	27/33 (81.8)	0.089^b^
Home	27/99 (27.3)	7/33 (21.2)	14/33 (42.4)	6/33 (18.2)	
Discharge medication					
Aspirin	99/99 (100.0)	33/33 (100.0)	33/33 (100.0)	33/33 (100.0)	..
ACEI/ARB	92/99 (92.9)	30/33 (90.9)	30/33 (90.9)	32/33 (97.0)	0.693^b^
Beta-blocker	91/99 (91.9)	31/33 (93.9)	30/33 (90.9)	30/33 (90.9)	0.999^b^
P2Y_12_ receptor inhibitor	96/99 (97.0)	31/33 (93.9)	32/33 (97.0)	33/33 (100.0)	0.771^b^
Statin	97/99 (98.0)	32/33 (97.0)	32/33 (97.0)	33/33 (100.0)	0.999^b^
Outcomes					
MACE at 1 year^f^	22/100 (22.0)	6/33 (18.2)	6/34 (17.6)	10/33 (30.3)	0.237^c^

Data are *n*/*N* (%) or median (IQR).

ACEI, angiotensin-converting enzyme inhibitors; ARB, angiotensin receptor blockers; CABG, coronary artery bypass grafting; GRACE, global registry of acute coronary events; LAD, left anterior descending coronary artery; LMWH, low-molecular-weight heparin; PCI, percutaneous coronary intervention.

^a^Kruskal–Wallis *H* test.

^b^χ^2^ test or Fisher’s exact test.

^c^Test for trend.

^d^Defined as lesions with ≥75% luminal stenosis.

^e^Defined as first stented coronary lesion.

^f^Defined as a composite measure of cardiac death, non-fatal myocardial infarction, or ischaemia-driven revascularization.

**Figure 5:**
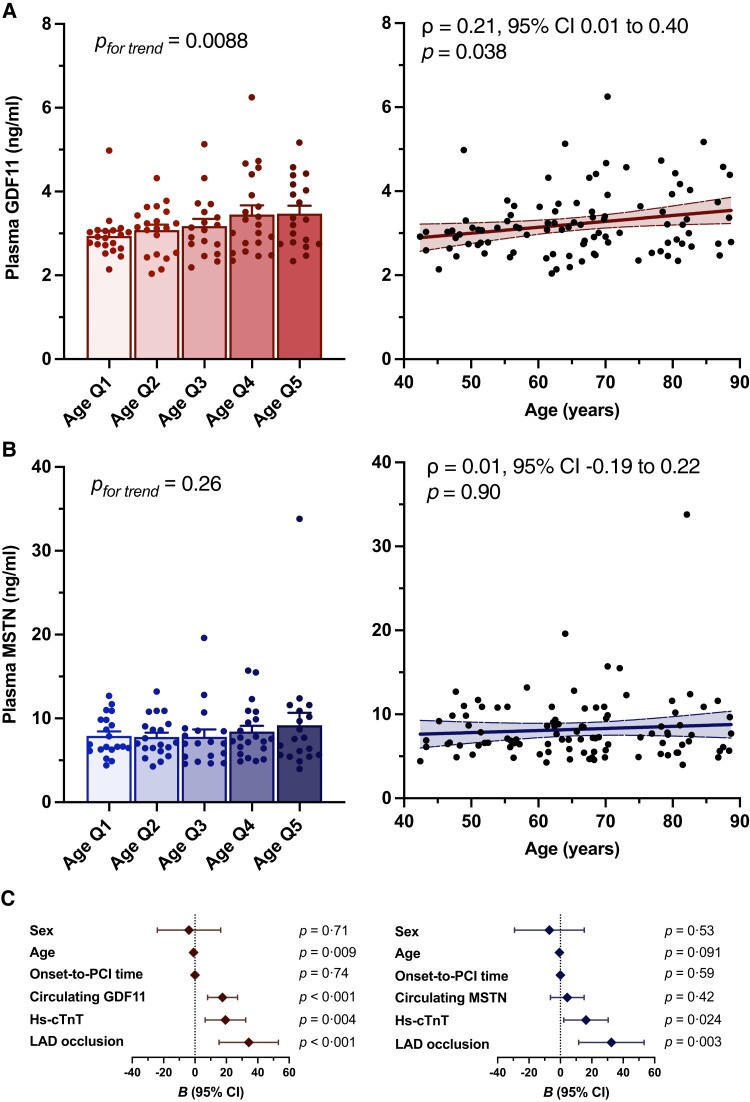
Age dependency of circulating GDF11/MSTN and their predictive value for the estimation of final infarct size in patients with acute myocardial infarction. (*A*, *B*) LC/LC-MS-based quantification of plasma GDF11 and MSTN per age quintile (*n* = 20/quintile; left) and Spearman rank correlation between continuous GDF11 and MSTN, respectively, with age (right). Simple linear regression and 95% confidence bands of the best fit line is plotted. (*C*) Unstandardized coefficients (*B*) of each independent variable included in the final regression model (model 3) ranked by their importance for the prediction of standardized peak CK-MB levels, a surrogate of final infarct size. Line length corresponds with the 95% CI. *P* values were calculated by one-way ANOVA [*A* (left), *B* (left), *C*] or Spearman rank correlation [*A* (right), *B* (right)], with Spearman’s ρ along with its 95% CI noted in the respective panel of *A* and *B*, respectively. Data in A and B (left panels) are presented as bar graphs with error bars (mean and SEM) with single data points superimposed.

When we stratified all patients according to age quintiles, a linear increase in circulating GDF11 was identified, with a positive monotonic relationship between age and continously coded circulating GDF11 protein levels (*Figure 5A*; Spearman’s ρ = 0.21, *P* = 0.038). In contrast, this association was abolished when MSTN was introduced as the independent variable (*Figure 5B*; Spearman’s ρ = 0.013, *P* = 0.90). Next, hierarchical regression models were built to test whether circulating GDF11 improves the prediction of infarct size over and beyond established risk factors, including sex, age, and LAD occlusion, as determined during initial coronary angiography, onset-to-PCI time, and high-sensitivity cardiac troponin (hs-cTnT), assessed at the time of the acute presentation. The model consisting of age, sex, LAD occlusion, onset-to-PCI time, and hs-cTnT to predict final infarct size, as estimated by standardized peak CK-MB levels,^72,73^ performed considerably well (*R*^2^ = 0.29, *P* < 0.001). Adding GDF11 to the model markedly improved the prediction of final infarct size (*R*^2^ = 0.42, Δ*R*^2^ = 0.13, *P* < 0.001) (see Supplementary material online, *Table S5*), whereas MSTN did not (*R*^2^ = 0.30, Δ*R*^2^ = 0.008, *P* = 0.42) (see Supplementary material online, *Table S6*). Indeed, with increasing GDF11 tertiles, an increase in standardized CK-MB levels of 17.55 [95% confidence interval (CI), 8.00–27.09 xURL, *P* < 0.001] was observed, whereas MSTN did not add to the prediction (4.36, 95% CI −6.36 to 15.09 xURL, *P* = 0.42) (*Figure 5C*).

## 4. Discussion

Formerly described as a promising heart and muscle rejuvenating factor,^27,28^ high systemic GDF11 failed to show beneficial effects on cardiac structure and function in more recent studies.^43,44^ In line with its originally reported capacity to repress cellular regeneration,^35^ our data support the notion that high levels of circulating GDF11 exert detrimental effects on the adult heart subjected to I/R. In fact, high levels of circulating GDF11 augmented apoptosis-mediated myocardial injury in mice irrespective of their age and were independently associated with increased infarct size in prospectively recruited patients with MI.

Albeit other forms of cell death determine myocardial infarct size,^10^ it is interesting to note that surrogates of inflammation and oxidative stress remained unaffected by rGDF11 supplementation in both young (*Figure 3B* and *C*) and aged (*Figure 3E* and *F*) mice subjected to I/R. Indeed, necroptosis and pyroptosis are featured by the loss of plasma membrane integrity and as such represent powerful triggers of inflammation. In contrast, cardiomyocyte apoptosis, an energy-consuming process regulated via intrinsic and extrinsic pathways,^11^ typically does not elicit an inflammatory reaction.^10^ With TUNEL staining of injured hearts pointing towards similar directions (*Figure 4A* and *B*), accelerated cardiomyocyte apoptosis appears to represent the main culprit of increased infarct size in rGDF11-supplemented animals.^10,74^ Interestingly, cytosolic mediators regulating cardiomyocyte survival pathways in the setting of I/R injury, such as Akt, Erk1/2, and Stat1,^10^ remained unaltered upon rGDF11 delivery (see Supplementary material online, *Figure S1*), implying distinct causative mechanisms: indeed, targeted transcriptomics together with immunomapping studies favour the involvement of non-myocyte CD105^+^ cell populations, with our *ex vivo* study’s hypothesis-generating results suggesting a possible indirect (paracrine-mediated) effect.^12^ However, current knowledge on the cardioprotective function of this type of cells relies predominantly on experimental work involving isolated/transplanted CD105^+^ cells (or cell products derived therefrom); thus, whether this concept is also applicable to endogenous (resident) CD105^+^ cells remains to be addressed by future experimental work using suitable *in vivo* models.

*Mstn* and *Gdf11* share many functional properties for muscle mass control in mice,^75^ but the contrasting myocardial expression pattern during ageing and upon I/R injury (*Figure 1B*)^59,60^ coupled with marked differences in their predictive utility for final infarct size in human patients (*Figure 5C*) strongly suggests that GDF11 and MSTN play distinct roles during I/R injury. In fact, both aptamer- and antibody-based detection methods commonly employed in the past were found to be non-specific for GDF11 (and thus to cross-react with MSTN): indeed, in contrast to the initial paradigm,^27,28^ various studies have reported variable or no significant age-related change in GDF11 protein levels in humans.^29–31^ Importantly, circulating MSTN protein levels were reported to exceed those of GDF11 in mice^29,33^ and humans^30,31^ as they did in the current study.

Hence, initially reported associations might have been driven, at least in part, by MSTN cross-reactivity. In this regard, it is noteworthy that elevated GDF11 + MSTN levels, as assessed by the above-noted assays, were linked to improved cardiovascular outcomes in patients with ischaemic heart disease^76^ and were recently shown to inversely associate with the composite outcome comprising non-fatal MI, non-fatal stroke, heart failure, and all-cause death in patients recruited across nine different studies and at high risk for adverse events using machine learning–based approaches.^77^ Although our study in human MI patients was not designed to gauge the association of high GDF11 or MSTN with major adverse cardiovascular events, it is interesting to note that the composite measure of cardiac death, non-fatal MI, or ischaemia-driven revascularization within 1 year tended to occur less likely with increasing MSTN levels (*p_trend_* = 0.076) (see Supplementary material online, *Table S4*), whereas no such association was observed with GDF11 (*Table 2*).

Collectively, our study’s results challenge the initially reported heart rejuvenating effects of GDF11 and suggest that high levels of circulating GDF11 exacerbate myocardial injury irrespective of age, with similar results obtained in prospectively recruited patients with MI. Hence, persistently high levels of circulating GDF11 during ageing may contribute to the age-dependent loss of endogenous cardioprotective mechanisms and thus poor outcomes of elderly patients post-MI.

### 4.1 Limitations

Considering the lack of significant effects of sex on circulating GDF11,^30^ only male C57BL/6 mice have been used in the current study. As sex-specific differences in the regulation of its closely related homolog MSTN exist,^78^ further studies may be warranted to test whether systemic GDF11 replenishment exerts similar effects on female hearts upon I/R injury. Second, although the abundancy of cardiomyocytes as the major cell type populating the adult heart^22^ and the unchanged inflammatory profiles favour increased apoptosis of cardiomyocytes as the main mechanism triggering differences in infarct size, accelerated cell death of non-myocyte cells may contribute as well. Third, although CD105 expression is mainly confined to mesenchymal/stromal cells with high myogenic differentiation potential such as cardiac-specific mesenchymal stromal cells,^13^ as confirmed by single-cell RNA-sequencing,^79^ we cannot exclude that other cell types, including fibroblasts, endothelial cells, or immune cells, also contribute to observed effects. In fact, while the precise cellular origin of CD105^+^ (and CD45^−^) cells remains elusive, their intrinsic cardiac origin is out of question^80^ most likely representing a heterogeneous, non-myocyte cell population,^81^ prompting us to gauge the effects of GDF11 using isolated CD105^+^ cells. Although targeted transcriptomics and immunomapping studies favour an indirect effect, our *ex vivo* study does not provide any direct experimental evidence, highlighting the need for further innovative studies to disentangle the mechanistic basis of resident CD105^+^ cells’ function during myocardial I/R injury. Finally, given the unavailability of cardiac magnetic resonance or single-photon emission computed tomography–derived measures of final infarct size in SPUM-ACS (ClinicalTrials.gov Identifier: NCT01000701), standardized peak CK-MB values were used as a surrogate thereof.

## Supplementary material

Supplementary material is available at *Cardiovascular Research* online.

## Authors’ contributions

S.K., C.B., D.V., A.A., Fa.M., and T.F.L. conceived the study; S.K., A.A., C.B., D.V., Fa.M., L.L., F.B., N.B., C.D.C., A.R., and F.C. performed the experiments; S.K., D.V., Fa.M., and A.A. analysed and interpreted the data; S.K., A.A., and T.F.L. wrote the manuscript. All co-authors revised the work critically for important intellectual content and approved the version to be published and agreed to be accountable for all aspects of the work in ensuring that questions related to the integrity of any part of the work presented are appropriately investigated and resolved.

## Supplementary Material

cvad153_Supplementary_Data

## Data Availability

All data supporting the findings reported herein will be shared upon reasonable request to the corresponding author.
