# Supplementary material for: Circulating GDF11 exacerbates myocardial injury in mice and associates with increased infarct size in humans
Source: Cardiovasc Res. 2023 Sep 23;119(17):2729–42. doi: 10.1093/cvr/cvad153 (PMC10757585; doi:10.1093/cvr/cvad153)
Supplement: cvad153_Supplementary_Data [file cvad153_supplementary_data.docx]

**Supplementary material**

**Circulating GDF11 exacerbates myocardial injury in mice and associates with increased infarct size in humans**

**Simon Kraler, Carolina Balbi, Daria Vdovenko, Tetiana Lapikova-Bryhinska, Giovanni G. Camici, Luca Liberale, Nicole Bonetti, Candela Diaz Canestro, Fabienne Burger, Aline Roth, Federico Carbone, Giuseppe Vassalli, Francois Mach, Shalender Bhasin, Florian A. Wenzl, Olivier Muller, Lorenz Räber, Christian M. Matter, Fabrizio Montecucco, Thomas F. Lüscher, Alexander Akhmedov**

**Table of contents**

**Pilot study** Hypothesis-generating results of the pilot study using isolated CD105^+^ cells

cells

**Key resources table** Table listing reagents and resources used in the present study

**Detailed methods** Detailed methods descriptions on tissue sampling and processing, targeted transcriptomics, the clinical study, and hierarchical regression models

**Figure S1** Cytosolic mediators of intrinsic cardioprotective pathways in rGDF11 and vehicle-treated mice stratified by age

**Figure S2** Validation of Nkx2-5 and Gata4 expression at mRNA and protein levels

**Figure S3** Nkx2-5 protein expression in CD105^-^ and CD105^+^ heart regions as assessed by double-antibody immunohistochemistry

**Figure S4** Caspase-3 expression in the pericellular region of CD105^+^ and CD105^-^ areas

**Figure S5** NKX2-5 and GATA4 expression in human cardiac

mesenchymal stromal cells exposed to high extracellular GDF11

**Figure S6** Pilot study

**Figure S7** Flow-chart of the study involving human patients with acute myocardial infarction recruited in SPUM-ACS

**Figure S8** Original (uncropped) immunoblot images

**Table S1** Ingenuity pathway analysis-guided transcriptomics

**Table S2** Baseline characteristics of SPUM-ACS participants stratified by GDF-11/MSTN measurement status

**Table S3** Baseline characteristics of SPUM-ACS participants with acute myocardial infarction stratified by MSTN tertiles.

**Table S4** Risk of adverse events, coronary lesion characteristics, discharge medication and 1-year MACE rates of all patients according to MSTN levels

**Table S5** Hierarchical multiple linear regression for the prediction of myocardial infarct-size in humans

**Table S6** Myocardial infarct-size, estimated by peak CK-MB, predicted by hierarchical multiple linear regression

**References**

**Pilot study**

Given the unavailability of a suitable experimental *in vivo* model to gauge the role of endogenous CD105^+^ cells during GDF11-mediated I/R injury, an *ex vivo* pilot study was performed. To that end, CD105^+^ cells were isolated and exposed to physiologically relevant concentrations of rGDF11 (**Supplementary material online, detailed methods**), showing Nkx2-5 expression patterns at both mRNA and protein levels that were intriguingly similar to those observed in cardiac tissues following I/R injury (**Supplementary material online, Figures S5-S6**). While the initial paradigm that CD105^+^ cells may replenish the pool of injured cardiomyocytes following myocardial injury was disproven by several lines of evidence,^1–5^ more recent evidence points toward indirect cardioprotective mechanisms involving the paracrine release of anti-apoptotic, immunomodulatory, and pro-angiogenic factors, with the non-coding miRNA-content of their payload being of particular relevance in the setting of I/R.^6,7^ Notably, a targeted miRNA screen of the CD105^+^ cell-derived secretome focusing on (patho-)physiological relevant miRNAs released by this cell type (miR-210, miR-132, miR-146)^6^ revealed alterations in the miRNA content, with a notable enrichment of miR-132 upon exposure to high extracellular GDF11 (**Supplementary material online, Figure S6B**), coinciding with a loss of the secretome’s cardioprotective effects (**Supplementary material online, Figure S6C**). Indeed, while media conditioned by CD105^+^ cells exposed to control conditions attenuated HL-1 cardiomyocyte apoptosis, in line with previous reports,^6,8^ the anti-apoptotic effect was completely abolished if media derived from rGDF11-treated CD105^+^ cells (and thus enriched in cardiac homeostasis perturbing miR-132^9–11^) was used. This preliminary finding is hypothesis-generating, given that miR-132 upregulation is mechanistically implicated in Foxo3 repression,^9^ a potent regulator of cardiomyocyte survival in the setting of I/R,^10^ with a phase 1b study utilizing CDR132L (i.e., antisense oligonucleotide targeting miR-132) showing promising results in patients with chronic heart failure of ischaemic origin.^11^

In summary, albeit further experimental studies are certainly warranted to test whether former^6–8,12–15^ and herein reported cardioprotective effects of CD105^+^ cells are also applicable to resident CD105^+^ cells, these data may suggest that high extracellular GDF11 induces transcriptional changes of non-myocyte CD105^+^ cells, coinciding with a loss of their secretome’s cardioprotective effects.

**Key resources table**

| **REAGENT or RESOURCE** | **SOURCE** | **IDENTIFIER** |
| --- | --- | --- |
| **Antibodies** |  |  |
| Ly-6G anti-mouse, dil: 1:100 | BD Pharmingen™ | [AB_2739207](http://antibodyregistry.org/AB_2739207) |
| CD68 anti-mouse, dil: 1:400 | Bio-Rad (Formerly AbD Serotec) | [AB_324217](http://antibodyregistry.org/AB_324217) |
| CD31 anti-mouse, dil.: 1:500 | Santa Cruz Biotechnology | [AB_2801330](http://antibodyregistry.org/AB_2801330) |
| SMA anti-mouse, dil.: 1:20 | Thermo Fisher Scientific Inc. | [AB_10979529](http://antibodyregistry.org/AB_10979529) |
| 4-HNE anti-mouse, 1 μg/ml | Oxis International Inc. | Cat# 24327 |
| Di bromo tyrosine (DiBrY) anti-mouse, 10 μg/ml | AMS biotechnology | Cat# AMS.MBY-020P |
| NKX2-5 anti-mouse, dil: 1:1000 | Abcam | [AB_10863313](http://antibodyregistry.org/AB_10863313) |
| GATA4 anti-mouse, dil: 1:1000 | Thermo Fisher Scientific Inc. | [AB_2547138](http://antibodyregistry.org/AB_2547138) |
| GAPDH anti-mouse, dil: 1:10’000 (tissue), dil:1:40’000 (cells) | Millipore Corporation | [AB_10615768](http://antibodyregistry.org/AB_10615768) |
| NKX2-5 anti-human, dil: 1:1000 | Abcam | [AB_10863313](http://antibodyregistry.org/AB_10863313) |
| GATA4 anti-human, dil: 1:1000 | Abcam | [AB_10670538](http://antibodyregistry.org/AB_10670538) |
| pAkt anti-mouse, dil: 1:1000 | Cell Signaling | [AB_329825](http://antibodyregistry.org/AB_329825) |
| Akt anti-mouse, dil: 1:1000 | Cell Signaling | [AB_329827](http://antibodyregistry.org/AB_329827) |
| Stat1 anti-mouse, dil: 1:1000 | Cell Signaling | [AB_2198300](http://antibodyregistry.org/AB_2198300) |
| p-p44/p42 MAPK anti-mouse, dil: 1:1000 | Cell Signaling | [AB_331646](http://antibodyregistry.org/AB_331646) |
| p44/p42 MAPK anti-mouse, dil: 1:1000 | Cell Signaling | [AB_330744](http://antibodyregistry.org/AB_330744) |
| CD105 anti-mouse, dil: 1:200 | Abcam | Cat #ab221675 |
| Nkx2-5 anti-mouse, dil: 1:150 | Novus Biologicals | [AB_11003900](http://antibodyregistry.org/AB_11003900) |
| Cleaved Caspase-3 anti-mouse, dil: 1:500 | Cell Signaling | [AB_2341188](http://antibodyregistry.org/AB_2341188) |
| **Biological Samples** |  |  |
| Mice serum | This paper | N/A |
| Mice cardiac tissue | This paper | N/A |
| Human EDTA-plasma samples | SPUM-ACS | ClinicalTrials.gov Identifier: NCT01000701  See REF^16,17^ |
| **Chemicals, Peptides, and Recombinant Proteins** |  |  |
| O2^-^-sensitive dye dihydroethidium (DHE) | Thermo Fisher Scientific | Cat# D11347 |
| DMSO | Sigma Aldrich | Cat# 67-68-5 |
| Human recombinant GDF11 | PeproTech | Cat# 120-11 AA sequence (monomer; sequence position 299-407): NLGLDCDEHS SESRCCRYPL TVDFEAFGWD WIIAPKRYKA NYCSGQCEYM FMQKYPHTHL VQQANPRGSA GPCCTPTKMS PINMLYFNDK QQIIYGKIPG MVVDRCGCS |
| Staurosporine | Sigma Aldrich | Cat# 62996-74-1 |
| TRIzol reagent | Thermo Fisher Scientific | Cat# 15596026 |
| Isoflurane | Piramal Pharma Limited | AP/DRUGS/220/96 |
| Ketamine-xylazine | Streuli | QN01AX03/QN05CM92 |
| Evans blue dye | Sigma Aldrich | Cat# 314-13-6 |
| Triphenyltetrazolium chloride | Sigma Aldrich | Cat# 298-96-4 |
| NaCl 0.9% Ecolav® | Braun | Cat# 3570350 |
| Formaldehyde 10% | Sigma Aldrich | Cat# F1635-1GA |
| Dulbecco’s medium (IMDM) | Lonza | Cat# 12-722F |
| Fetal bovine serum (FBS) | Gibco, Life Technologies | Cat# 11573397 |
| Penicillin-streptomycin, 1% | Gibco, Life Technologies | Cat# 15-140-122 |
| Claycomb Medium | Sigma-Aldrich | Cat# 51800C |
| Norepinephrine, 100 μM | Gibco, Life Technologies | Cat# 51-41-2 |
| L-glutamine, 4 mM | Gibco, Life Technologies | Cat# 11539876 |
| 1% Triphenyltetrazolium chloride (TTC) diluted in phosphate buffer (pH 7.4) | Millipore | Cat# 298-96-4 |
| **Critical Commercial Assays** |  |  |
| Dead End™ colorimetric terminal deoxynucleotidyl-transferase-mediated dUTP nick end labeling (TUNEL) system | Promega | Cat# G7360 |
| Mouse cardiac troponin-I ELISA | Life Diagnostics Inc. | Cat# CTNI-1-HS |
| Mouse CXCL1/KC DuoSet ELISA | R&D Systems | Cat# DY453 |
| Mouse CCL2/JE/MCP-1 DuoSet ELISA | R&D Systems | Cat# DY479-05 |
| Cellstain™ Double Stain Kit | Dojindo EU | Cat# CS01-10 |
| LC-MS/MS-based GDF11/MSTN assay | This paper | See REF^18^ |
| RT^2^ First Strand Kit | Qiagen | Cat# 330404 |
| RT^2^ SYBR Green ROX qPCR Master mix | Qiagen | Cat# 330520 |
| TaqMan^TM^ Gene Expression Master Mix | Thermo Fischer Scientific | Cat# 4369016 |
| Pierce™ BCA Protein Assay Kit | Thermo Scientific™ | Cat# 23225 |
| QuantiMir™ Kit | System Biosciences, Inc. | Cat # RA420A-1 |
| Primary Antibody Diluent | Diagnostic Biosystems | Cat# K004 |
| BOND Epitope Retrieval Solution 2 | Leica Biosystems | Cat# AR9640 |
| Novocastra Protein Block | Leica Biosystems | Cat# RE7102 |
| BOND Polymer Refine Red Detection | Leica Biosystems | Cat# DS9390 |
| BOND Primary Antibody Diluent | Leica Biosystems | Cat# AR9352 |
| BOND Polymer Refine Detection | Leica Biosystems | Cat# DS9800 |
| **Experimental Models: Organisms/Strains** |  |  |
| Male C57BL/6 mice | Janvier Labs (France) | N/A |
| **Experimental Models: Cell lines** |  |  |
| Human cardiac mesenchymal stromal cells | This paper | See REF^6,8,19,20^ |
| HL-1 cardiomyocyte cell line | LSU Health Sciences Center | See REF^6,8^ |
| **Oligonucleotides** |  |  |
| Gapdh TaqMan probe | Thermo Fisher Scientific | Cat# Mm99999915_g1 |
| Gdf11 TaqMan^TM^ probe | Thermo Fisher Scientific | Cat# Mm01159973_m1 |
| MstnTaqMan^TM^ probe | Thermo Fisher Scientific | Cat# Mm01254559_m1 |
| Nkx2-5 TaqMan^TM^ probe | Thermo Fisher Scientific | Cat# Mm00657783_m1 |
| Gata4 TaqMan^TM^ probe | Thermo Fisher Scientific | Cat# Mm00484689_m1 |
| miR16 SYBR Green probe | Microsynth AG | Seq: uagcagcacguaaauauuggcg |
| miR132-3p SYBR Green probe | Microsynth AG | Seq: uaacagucuacagccauggucg |
| miR146-5p SYBR Green probe | Microsynth AG | Seq: ugagaacugaauuccauggguu |
| miR210-3p SYBR Green probe | Microsynth AG | Seq: cugugcgugugacagcggcuga |
| **Software and Algorithms** |  |  |
| ImageJ 1.50 | National Institutes of Health / Laboratory for Optical and Computational Instrumentation | [See](https://imagej.nih.gov/ij/) REF^21^ |
| Fiji v.2.9.0 | National Institutes of Health / Laboratory for Optical and Computational Instrumentation | See REF^22^ |
| MetaMorph v6.0 | Molecular Devices - Universal Imaging Corporation | <http://www.moleculardevices.com/systems/metamorph-research-imaging/metamorph-microscopy-automation-and-image-analysis-software> |
| GraphPad Prism | Graphpad | <https://www.graphpad.com/> |
| SPSS statistics 28.0 | IBM Corporation | <https://www.ibm.com/analytics/spss-statistics-software> |
| R version 4.2.1 | R Foundation for Statistical Computing | <https://www.R-project.org/> |
| **Other** |  |  |
| Chow diet (mouse studies) | Kliba Nafag | Cat# 3802 |
| PROLENE® Polypropylene Suture 8-0 | Ethicon | Cat# 8741H |
| Cell culture flask (75 cm) | Sigma-Aldrich | Cat# SIAL0641 |
| BOND-III Fully Automated IHC and ISH Staining System | Leica Biosystems | Cat#21.2201 |

**Detailed methods**

- 1. **Tissue sampling and processing**

In the first cohort of animals (n = 5/group), young (3-4 months), middle-aged (12-14 months) and aged (22-24 months) mice were euthanized by i.p. injection of ketamine-xylazine (total dose of 4 mg) followed by terminal bleeding. After performing medial sternotomy, the heart and aorta were rapidly excised, rinsed with ice-cold PBS, immediately snap-frozen, and stored at -80°C until further processing. A subgroup of aged animals was also subjected to I/R, following the same protocol as detailed in section 2.1, with age-matched mice serving as controls.

In a second cohort of mice undergoing myocardial I/R after 30 days of rGDF11 or vehicle treatment, animals were anesthetized with isoflurane (4-5% for induction and 1-2% for maintenance) following the indicated reperfusion periods, their chest reopened and the LAD re-occluded, upon which they were sacrificed by i.p. injection of ketamine-xylazine (total dose of 4 mg). Final infarct size was determined by injecting Evans blue dye (2%; Sigma) to visualize the area at risk (AAR). Next, the heart was rapidly excised, rinsed with saline solution and frozen until further processing. Frozen cardiac tissues were thinly sectioned transversely from the heart’s apex to its base (5-6 slices per heart). In the presence of 1% triphenyltetrazolium chloride (TTC) diluted in phosphate buffer (pH 7.4), these 2 mm heart sections were then incubated at 37°C for 15 min, upon which they were fixed in 10% formaldehyde solution for 24 hours. High-resolution tissue images (Nikon, Tokyo, Japan) were taken by an independent investigator blinded to each mouse’ group allocation and imported into MetaMorph 6.0 (Molecular Devices - Universal Imaging Corporation) to allow for precise delimitation of continuously perfused (blue), stained ischaemic viable (red) and unstained necrotic tissue (white). AAR and ventricular infarct zone (I) were normalized to total ventricular surface area (V) and are expressed as AAR/V and I/V, respectively.

To allow for immunostaining, hearts from animals sacrificed after 24h of reperfusion were embedded in optimal cutting temperature compound (OCT, Tissue-Tek; Sakura, Japan), cut serially from the occlusion locus to the apex in 7 μm sections, and 5 midventricular sections per animal were stained for neutrophils (Ly-6G anti-mouse, dilution 1:100; BD Pharmingen™, San Jose, CA, USA), macrophages (CD68 anti-mouse, dilution: 1:400; ABD Serotec, Dusseldorf, Germany), endothelial (CD31 anti-mouse, dilution 1:500, Santa Cruz Biotechnology, Santa Cruz, CA, USA), smooth muscle cells (SMA anti-mouse, dilution 1:20, Thermo Scientific Inc., Waltham, MA, USA), the O_2_^-^-sensitive dye dihydroethidium (DHE, Molecular Probes, Life Technologies Corporation, Zug, Switzerland), the lipid membrane peroxidation product 4-hydroxy-2-nonenal (mouse anti-4-HNE, 1 μg/ml, Oxis International Inc, Foster City, CA, USA) and the 3,5-dibromotyrosine (Di bromo tyrosine anti-mouse, 10 μg/ml, AMS biotechnology, LTD, Abingdon, UK). As above, MetaMorph 6.0 was used for quantification, with results presented as number of infiltrating cells per mm^2^ of total heart surface area, as reported in detail previously. To determine the number of cells undergoing nuclear fragmentation (a hallmark of apoptosis), we performed a TUNEL assay on OCT-embedded heart sections, using an *in-situ* apoptosis detection kit (DeadEnd™ Colorimetric TUNEL System, Promega, Madison, USA), in which diaminobenzidine served as the chromogenic substrate.

In all animals of cohort II and subjected to rGDF11/vehicle treatment and subsequent I/R injury, blood was collected at the time of sacrifice by cardiac puncture using serum collection tubes (Becton, Dickinson and Company, Franklin Lakes, NJ, USA), stored at room temperature in an upright position, before they were centrifuged at 4’500x g within 30 minutes and serum eventually transferred to pre-cooled cryotubes, that were subsequently stored at −80°C until further analysis.

- 1. **Targeted transcriptomics**

Hearts of animals subjected to 30 days of rGDF11 or vehicle treatment and subsequent I/R injury with 8 hours of reperfusion (aged 3-4 months; 5 animals/group) were freshly dissected, rinsed with ice-cold PBS, immediately snap-frozen in liquid N_2_ and stored at -80°C until further processing. Frozen hearts were crushed with a mortar and pestle, and total RNA was isolated with TRIzol (Invitrogen, Life Technologies Corporation, Zug, Switzerland). We used NanoDrop® ND1000 (NanoDrop Technologies, Wilmington, DE, USA) to assess concentration, purity, and integrity of isolated RNA, before creating a cDNA library (RT^2^ First Strand Kit, QIAGEN, Hilden, Germany). Next, we employed Ingenuity Pathway Analysis (IPA; version 36601845; <https://digitalinsights.qiagen.com/IPA>; Qiagen, Venlo, Netherlands) on the QIAGEN Knowledge Database, a highly structured database that encompasses more than 13 million individual datasets retrieved from proprietary, open source, and licensed biological content that has been aggregated, integrated, and manually curated, to identify downstream effectors of GDF11 relevant to the current study.^23,24^ This approach yielded a total of 60 protein-coding genes of which 4 were previously described downstream targets of GDF11 (*Cdkn1b, Nppa, Nppb, Atp2a2*), 6 linked to Smad2/3 proteins in the myocardium (*Ccl2, Tnf, Col3a1, Id2, Mycn, Ccnd1*), 40 associated with Smad2/3 proteins and linked to cardiovascular diseases (*Acta2, Apoa1, Bbc3, Becn1, Ccl3l3, Cdk4, Crp, Csf1, Ctgf, Esr1, Flt1, Fstl3, Hmox1, Ifng, Il2, Il6, Il1b, Il6r, Mdm2, Mmp9, Mtor, Nkx2-5, Nos2, Npr2, Pdgfb, Plau, Ppard, Ppargc1a, Ptgs2, Ptx3, Rac1, Rassf1, Serpine1, Tf, Tgfb1, Timp1, Timp3, Vegfa, Xiap*), and 11 associated with myocardial ischemia and/or reperfusion injury (*Cxcl2, Clu, Edn1, Eif2a, Eng, Mapk8, Nos3, Prkcb, Rhoa, Tnfsf11, Tnfrsf11*).^25–28^ Next, we performed RT-qPCR by using the RT^2^ SYBR Green ROX qPCR Master mix and 384-well plates (both QIAGEN, Hilden, Germany) precoated with predesigned primers specific for the candidate genes noted above. Gene expression data were analysed using the comparative CT method,^29^ with *Gapdh* serving as the reference gene. Genes with normalized counts greater than log_2_(FC) > 1.2 and FDR < 0.1 ^30^ with respect to controls were considered as differentially expressed.

- 1. **Cell lines**

CD105^+^ non-myocyte cells were isolated from right atrial appendage specimens, freshly obtained from patients undergoing surgical heart valve replacement without concomitant coronary artery disease, as reported previously.^6,8,19,20^ To that end, a primary *ex vivo* culturing technique was employed.^8^ Briefly, freshly obtained atrial specimens were rinsed with NaCl, immediately transferred to the laboratory, and cultured in basic Iscove’s modified Dulbecco’s medium (IMDM) supplemented with 20% fetal bovine serum (FBS) and 1% v/v penicillin-streptomycin. Within 14 days of *ex vivo* tissue culture, the cellular outgrowth was collected and seeded into fibronectin-coated 75 cm cell culture flasks, yielding a monolayer of non-myocyte cells that was gathered, purified, and subsequently used for the *in vitro* experiments. Phenotypic, functional and antigenic profiles of these cells have been reported previously (formerly referred to as cardiac mesenchymal progenitor cells given their multilineage potential),^6,8^ with this non-myocyte cell type showing marked enrichment of mesenchymal/stromal markers, such as CD105, CD90 and CD13, while being typically negative for CD45. The study was approved by the local Ethical Committee and performed according to the Declaration of Helsinki. All patients gave written informed consent to the collection of tissues. Mouse HL-1 cardiomyocytes were obtained from the LSU Health Sciences Center (New Orleans, LA, USA), and cultured as reported previously.^6^ To ensure cellular maintenance and mature cardiomyocyte behaviour,^31^ HL-1 cardiomyocytes were grown in Claycomb Medium supplemented with 100 μM norepinephrine, 10% FBS, 1% v/v penicillin streptomycin, and 4 mM L-glutamine. All cells were grown in a 5% CO_2_ atmosphere at 37°C.

- 1. ***In vitro* experiments and cell viability testing**

CD105^+^ (cardiac-specific mesenchymal stromal) cells were cultured in basal medium supplemented with rGDF11 (200 pmol/L) or vehicle (0.1% BSA containing PBS) over 5 days, upon which the medium was replaced by serum-free DMEM supplemented with 5 mM glucose, according to previously published protocols.^32,33^ At day 10, conditioned media (CM) was collected, centrifuged at 3000x g for 15 min, filtered through a 0.2 µm membrane (BD Biosciences), and protein concentration was quantified by BCA (Thermo Scientific™ Pierce™ BCA Protein Assay Kit). In a pilot study, changes in the non-coding miRNA-content of their secretome were studied focusing on three miRNAs (miR132-3p, miR146-5p and miR210-3p) previously shown to be functionally relevant in the setting of I/R.^6^ To evaluate CM-mediated cardioprotection, we then exposed HL1-cardiomyocytes to CM derived from either rGDF11- or vehicle-treated CD105^+^ non-myocyte cells before apoptosis was induced by staurosporine, as reported previously.^8^ To that end, HL1-cells were plated in 96-well plates (7×10^4^ cells/cm^2^), followed by apoptosis induction by 1μM of staurosporine (Sigma-Aldrich) in the presence of CM (50ug total protein/ml) derived from either rGDF-11- (rGDF11-CM) or vehicle-treated CD105^+^ non-myocyte cells (CTRL-CM). After 12 hours, cardiomyocytes were stained using the Cellstain™ Double Stain Kit (Dojindo EU, München, Germany) for 30 min at 37 °C, and cell viability was determined by quantification of DRAQ7 positive cells using a fluorescence microscope.

- 1. **miRNA analyses**

Transcriptional profiling of miRNAs focusing on non-coding RNA molecules enriched in the cMSC-derived secretome was done as previously described.^6^ Briefly, total RNA was isolated from conditioned media (CM) obtained from rGDF11- or PBS-treated cMSCs (n=6/group) using a TRIzol-based isolation protocol (Invitrogen, Life Technologies Corporation, Zug, Switzerland) and reverse transcribed leveraging Quanti-miR^TM^ kits (System Biosciences Inc., Palo Alto, CA) involving a multistep approach, as we have reported.^6^ Real-time analysis was performed on the CFX connect Bio-Rad Real-time PCR detection system using advanced SYBR Green kits (Bio-Rad, Hercules, CA, US) and miR16, miR132-3p, miR146-5p and miR210-3p specific primers. Quantification was done using the comparative CT method, with miR16 serving as the reference.^29^

- 1. **Clinical study**

Patients, 18 years or older, presenting to any of the four major university hospitals in Switzerland with a main diagnosis of acute coronary syndromes [ACS; ST-segment elevation MI (STEMI) or non ST-segment elevation ACS (NSTE-ACS)] and one of the following characteristics, (i) persistent ST-segment elevation or depression, T inversion or dynamic ECG changes, new left bundle branch block (LBBB), or (ii) evidence of positive troponin by local laboratory reference values with a rise and/or fall in serial troponin levels, or (iii) known coronary artery disease, specified as status after MI, CABG, or PCI or newly documented ≥50% stenosis of an epicardial coronary artery during the initial catheterization, were eligible for inclusion. Patients with severe physical disability, dementia (inability to comprehend study), or a life expectancy (for non-cardiac reasons) of less than 1 year were not eligible to participate in the study. During the early hospitalization period (12 to 24 hours after primary PCI), CK-MB values were measured serially at each institution and peak CK-MB values were documented, as reported previously.^34^ After discharge, all patients were scheduled for follow-up visits at 30 days (phone call) and at 1 year (clinical visit). The prespecified primary endpoint, defined as the composite measure of cardiac death, MI or ischemia-driven revascularization, was adjudicated by an external clinical endpoint committee comprising three certified cardiologists blinded to patient’s baseline characteristics using pre- specified adjudication forms. Individual peak CK-MB levels were standardized to each laboratory’s upper reference limit (URL) [CK-MB values (ng/ml) x URL], as suggested by the Joint ESC/ACCF/AHA/WHF Task Force for the Redefinition of Myocardial Infarction.^35^ High-sensitivity cardiac troponin (hs-cTnT) levels were measured centrally in the core laboratory, as reported previously.^16^ Based on Fisher’s z-transformation and normal approximation, we estimated that 89 randomly selected patients would be required to give the study 85% power to detect a Spearman correlation coefficient of 0.35 between standardized CK-MB levels and plasma GDF11 and MSTN levels, respectively, at a Bonferroni-adjusted significance level of 0.05 (two-sided). Assuming 10-15% of missing data, the final study population totalled 100 patients, drawn at random from the total study population of all 4 787 ACS patients (**Supplementary material online, Figure S7** and **Tables S2-S4**). Data reporting follows the STROBE initiative.^36^

- 1. **Hierarchical regression models**

Given the multicentre design of SPUM-ACS, individual peak CK-MB levels were standardized to the upper reference limit (URL) of the local laboratory [CK-MB values (ng/ml)/URL], as suggested by the Joint ESC/ACCF/AHA/WHF Task Force for the Redefinition of Myocardial Infarction.^35^ To study the association of standardized peak CK-MB and plasma GDF11 and MSTN, respectively, Spearman rank correlation coefficients were calculated. Informed by data- and theory-driven considerations,^18,37–41^ we then built step-wise multiple linear regression models, in which standardized peak CK-MB [x URL] served as the dependent (i.e., surrogate of infarct size),^40,41^ and sex, age, hs-cTnT, presence of LAD occlusion (i.e., proxy for anterior-wall infarction), symptom-to-PCI time (to control for CK-MB dynamics) and GDF11 or MSTN plasma levels as independent variables. Lack of independence (autocorrelation) between residuals was tested by the Durbin-Watson test. The linearity assumption of independent variables included in the final model, collectively and for each variable individually, was tested by plotting the studentized residuals against the unstandardized predicted values (final model) and by generating partial regression plots (for each predictor individually). If this assumption was not met, data were either log-transformed (hs-cTnT) or coded as categorical variables (GDF11 and MSTN, respectively). Homoscedasticity was assessed by visual inspection. To test for multicollinearity, we considered previously published thresholds,^42^ and calculated Pearson correlation coefficients between independent variables (each below <0·5) and the final model’s VIF. We checked for unusual data points by inspecting standardized residuals (outliers), estimating individual leverage values (high leverage points) and calculating Cook’s Distance using established thresholds.^43,44^ Finally, normal distribution of residuals was tested by plotting the expected against observed cumulative probability (P-P plot). The above-noted assumptions were met for all independent variables included in the final model, i.e., sex (female/male), age (years), log-transformed hs-cTnT (ng/ml), LAD occlusion (yes/no), symptom-to-PCI time (min), and tertiles of GDF11 or MSTN. Overall model fit was assessed by a variety of measures, including the multiple correlation coefficient R as well as the coefficient of determination (adjusted) R^2^ (**Supplementary material online, Tables S5-S6**), and overall model significance was tested using ANOVA F-test, with the threshold of α set at 0.05. All analyses were performed using R version 4.2.1 (R Foundation for Statistical Computing, Vienna, Austria), SPSS version 28.0 (IBM, Armonk, NY, USA) GraphPad Prism 8 (GraphPad Software, LLC, Massachusetts, USA).

***Figure S1.* Cytosolic mediators of intrinsic cardioprotective signalling pathways in rGDF11 and vehicle-treated mice stratified by age. A, B**: Total Akt (protein kinase B) expression and phosphorylation status (Akt-Ser473) of young and aged mice with (rGDF11) or without (CTRL) rGDF11 pre-treatment, respectively. **C, D**: Total p42/44 MAPK expression and phosphorylation status (p42-Tyr204 and p44-Thr202, respectively) of young and aged mice, respectively, receiving rGDF11 or vehicle treatment prior to I/R. **E, F**: Total protein expression of Stat1 following I/R injury in young and aged mice of both groups is shown. Note that representative immunoblots are shown in the bottom panel with C signifying mice receiving vehicle- and G rGDF11-treatment. Data are presented as bar graphs and error bars (mean and SEM) with single data points superimposed.

***Figure S2.*** Validation of Nkx2-5 and Gata4 expression at mRNA (A) and protein levels (B). Representative immunoblots are shown at the bottom of the figure. C denotes control and G rGDF11-treated mice. Data are presented as bar graphs with error bars (mean and SEM) with single data points superimposed.

***Figure S3.*** Nkx2-5 protein expression in CD105^-^ and CD105^+^ heart regions following I/R in mice with (rGDF11) or without (CTRL) rGDF11 pre-treatment, as assessed by double-antibody immunohistochemistry. Quantifications of Nkx2-5 protein expression in CD105^+^ and CD105^-^ regions are provided in panel A, whereas representative immunohistochemistry images are shown in panel B. Data are presented as bar graphs with error bars (mean and SEM) with single data points superimposed.

***Figure S4.*** Caspase-3 expression in the pericellular region of CD105^+^ and CD105- areas following I/R in mice subjected to rGDF11 (rGDF11) or control treatment (CTRL), quantified by double-antibody immunohistochemistry, with the pericellular region defined as an area extending 9x9 pixels, with 1 pixel equalling 1 µm. Data are presented as bar graphs with error bars (mean and SEM) with single data points superimposed.

******

***Figure S5.* *NKX2-5* and *GATA4* expression in human cardiac mesenchymal stromal cells exposed to high extracellular GDF11.** Panel A depicts mRNA expression of both *NKX2-5* and *GATA4*, while B shows GATA4 protein expression levels. Data are shown as violin plots (median and IQR) (A) or bar graphs (mean and SEM) (B) with single data points superimposed.

***Figure S6.* Pilot study: Isolated CD105^+^ cardiac cells exposed to high GDF11 phenocopy reductions in cardiac NKX2-5 protein expression, enrich miR-132 in their secretome, and lose their cardioprotective effects.** (A), Protein expression of NKX2-5 in CD105^+^ cardiac mesenchymal stromal cells after 5-day incubation with rGDF11 (200 pmol/L) or vehicle (CTRL). (B), Focused miRNA analysis of the CD105^+^ cell-derived secretome in conditions of high extracellular GDF11 relative to CTRL treatment. (C), Cardiomyocyte viability testing after 4-day exposure to media conditioned by CD105^+^ cells exposed to rGDF11 or CTRL treatment, with enrichment of miR-132 in the former. Representative immunofluorescence images are shown on the right. Apoptosis was induced by 1μM Staurosporine. *P*-values were calculated by unpaired Student’s t-test (A, B (top)], or (C) one-way ANOVA followed by Bonferroni post-hoc test. Data are presented as bar graphs with error bars (mean and SEM) with single data points superimposed.

***Figure S7.* Flow-chart of the study involving human patients with acute myocardial infarction recruited in SPUM-ACS.** Among the 4787 patients recruited in SPUM-ACS (ClinicalTrials.gov Identifier: NCT01000701), 100 randomly chosen individuals were included in the current study.

***Figure S8.* Original (uncropped) immunoblot images.** Note that representative images are highlighted in colour (red rectangle, Gata4; blue rectangle, Nkx2-5; green rectangle, Gapdh).

| **Gene name** | **Ingenuity-pathway analysis (IPA)** | **LOG_2_(FC)** | ***q-*value** |
| --- | --- | --- | --- |
|  |  |  |  |
| Acta2 | CVD-associated Smad2/3 protein | -0.1968 | 0.6252 |
| Apoa1 | CVD-associated Smad2/3 protein | 0.5837 | 0.1481 |
| Atp2a2 | GDF11 downstream target | -0.0268 | 0.9469 |
| Bbc3 | CVD-associated Smad2/3 protein | -0.4318 | 0.2842 |
| Becn1 | CVD-associated Smad2/3 protein | -0.1006 | 0.8027 |
| Ccl2 | Myocardial Smad2/3 protein | -0.6014 | 0.1363 |
| Ccl3 | CVD-associated Smad2/3 protein | -0.5353 | 0.1846 |
| Ccnd1 | Myocardial Smad2/3 protein | -0.1862 | 0.6439 |
| Cdk4 | CVD-associated Smad2/3 protein | -0.1653 | 0.6815 |
| Cdkn1b | GDF11 downstream target | -0.0245 | 0.9515 |
| Clu | Myocardial I/R-injury associated target | 0.0203 | 0.9598 |
| Col3a1 | Myocardial Smad2/3 protein | -0.3607 | 0.3708 |
| Crp | CVD-associated Smad2/3 protein | ·· | ·· |
| Csf1 | CVD-associated Smad2/3 protein | ·· | ·· |
| Ctgf | CVD-associated Smad2/3 protein | -0.1986 | 0.6220 |
| Cxcl12 | Myocardial Smad2/3 protein | 0.1032 | 0.7978 |
| Edn1 | Myocardial Smad2/3 protein | 0.0455 | 0.9100 |
| Eif2a | Myocardial Smad2/3 protein | -0.1087 | 0.7873 |
| Eng | Myocardial Smad2/3 protein | -0.2517 | 0.5321 |
| Esr1 | CVD-associated Smad2/3 protein | 0.0896 | 0.8239 |
| Flt1 | CVD-associated Smad2/3 protein | 0.1789 | 0.6569 |
| Fstl3 | CVD-associated Smad2/3 protein | -0.1976 | 0.6237 |
| Hmox1 | CVD-associated Smad2/3 protein | -0.2842 | 0.4805 |
| Id2 | Myocardial Smad2/3 protein | -0.3433 | 0.3943 |
| Ifng | CVD-associated Smad2/3 protein | ·· | ·· |
| IL1b | CVD-associated Smad2/3 protein | -0.4462 | 0.2685 |
| Il2 | CVD-associated Smad2/3 protein | ·· | ·· |
| Il6 | CVD-associated Smad2/3 protein | -0.4000 | 0.3211 |
| Il6ra | CVD-associated Smad2/3 protein | -0.1742 | 0.6654 |
| Mapk8 | Myocardial Smad2/3 protein | 0.1007 | 0.8025 |
| Mdm2 | CVD-associated Smad2/3 protein | -0.0620 | 0.8777 |
| Mmp9 | CVD-associated Smad2/3 protein | -0.5419 | 0.1792 |
| Mtor | CVD-associated Smad2/3 protein | -0.3478 | 0.3881 |
| Mycn | Myocardial Smad2/3 protein | -0.6692 | 0.0976 |
| Nkx2-5 | CVD-associated Smad2/3 protein | -1.6230 | 0.0010 |
| Nos2 | CVD-associated Smad2/3 protein | -0.0969 | 0.8099 |
| Nos3 | Myocardial Smad2/3 protein | 0.0867 | 0.8295 |
| Nppa | GDF11 downstream target | 0.6339 | 0.1164 |
| Nppb | GDF11 downstream target | -0.4775 | 0.2364 |
| Npr2 | CVD-associated Smad2/3 protein | 0.0388 | 0.9232 |
| Pdgfb | CVD-associated Smad2/3 protein | 0.3195 | 0.4278 |
| Plau | CVD-associated Smad2/3 protein | -0.4597 | 0.2543 |
| Ppard | CVD-associated Smad2/3 protein | -0.1693 | 0.6742 |
| Ppargc1a | CVD-associated Smad2/3 protein | -0.6974 | 0.0843 |
| Prkcb | Myocardial Smad2/3 protein | -0.4299 | 0.2863 |
| Ptgs2 | CVD-associated Smad2/3 protein | 0.3878 | 0.3361 |
| Ptx3 | CVD-associated Smad2/3 protein | -0.5371 | 0.1831 |
| Rac1 | CVD-associated Smad2/3 protein | -0.0575 | 0.8865 |
| Rassf1 | CVD-associated Smad2/3 protein | 0.3210 | 0.4256 |
| Rhoa | Myocardial Smad2/3 protein | -0.0845 | 0.8338 |
| Serpine1 | CVD-associated Smad2/3 protein | 0.0893 | 0.8244 |
| Tgfb1 | CVD-associated Smad2/3 protein | -0.1315 | 0.7441 |
| Timp1 | CVD-associated Smad2/3 protein | -0.0068 | 0.9865 |
| Timp3 | CVD-associated Smad2/3 protein | 0.1685 | 0.6757 |
| Tnf | Myocardial Smad2/3 protein | -0.9054 | 0.0254 |
| Tnfrsf1 | Myocardial Smad2/3 protein | -0.1058 | 0.7927 |
| Tnfsf1 | Myocardial Smad2/3 protein | -0.9353 | 0.0210 |
| Trf | CVD-associated Smad2/3 protein | -0.2108 | 0.6007 |
| Vegfa | CVD-associated Smad2/3 protein | 0.0413 | 0.9184 |
| Xiap | CVD-associated Smad2/3 protein | -0.0611 | 0.8795 |

***Table S1.* Ingenuity pathway analysis-guided transcriptomics.** In the first column, genes identified within the *QIAGEN Knowledge Base*^23,24^ are listed. Fold change (FC) corresponds to the comparison of rGDF11 vs. CTRL mice (n = 5/group).

|  | **All patients** | **GDF11/MSTN assessed** | **GDF11/MSTN not assessed** | ***p*-value** |
| --- | --- | --- | --- | --- |
|  | **n = 4 787** | **n = 100** | **n = 4 687** |  |
| Age (years) | 63.5 (54.3-73.0) | 66.4 (56.0-77.9) | 63.5 (54.2-73.0) | 0.077 |
| Female | 986/4787 (20.6) | 23/100 (23.0) | 963/4687 (20.5) | 0.550 |
| BMI (kg/m^2^) | 26.6 (24.2-29.4) | 26.7 (24.2-29.1) | 26.6 (24.2-29.4) | 0.800 |
| H_x_ of dyslipidaemia | 3021/4785 (63.1) | 57/100 (57.0) | 2964/4685 (63.3) | 0.200 |
| H_x_ of hypertension | 2698/4785 (56.4) | 53/99 (53.5) | 2645/4686 (56.4) | 0.560 |
| H_x_ of systemic inflammatory disease | 128/4787 (2.7) | 3/100 (3) | 125/4687 (2.7) | 0.750 |
| Hs-CRP (mg/l) | 2.7 (1.1-7.5) | 2.5 (1.1-9.5) | 2.7 (1.1-7.4) | 0.520 |
| Hs-cTnT (ng/l) | 197 (59-650) | 246 (67-713) | 196 (59-646) | 0.240 |
| NT-proBNP (ng/l) | 341 (109-1200) | 331 (113-1363) | 341 (109-1195) | 0.680 |
| eGFR (ml/min/1.73m^2^) | 87.8 (71.7-98.9) | 86.7 (72.9-100.1) | 87.8 (71.7-98.8) | 0.680 |
| Baseline medication |  |  |  |  |
| Aspirin | 1366/3087 (44.3) | 23/52 (44.2) | 1343/3035 (44.3) | >0.999 |
| P2Y_12_ inhibitor | 358/3086 (11.6) | 5/52 (9.6) | 353/3034 (11.6) | 0.830 |
| ACE inhibitor | 709/3064 (23.1) | 14/52 (26.9) | 695/3012 (23.1) | 0.620 |
| Diuretic | 764/3081 (24.8) | 11/52 (21.2) | 753/3029 (24.9) | 0.540 |
| Statin | 1283/3080 (41.7) | 19/52 (36.5) | 1264/3028 (41.7) | 0.450 |

***Table S2.* Baseline characteristics of SPUM-ACS participants stratified by GDF-11/MSTN measurement status.** Data are n/N (%), or median (IQR). BMI denotes body mass index, WBC white blood count, hs-CRP high-sensitivity C-reactive protein, LDL-C low-density lipoprotein cholesterol, hs-cTnT high-sensitivity cardiac troponin T, NT-proBNP N-terminal prohormone brain natriuretic peptide, eGFR estimated glomerular filtration rate, P2Y_12_ P2Y_12_ receptor for adenosine diphosphate, and ACE, angiotensin-converting enzyme.

|  | **All Patients** | **MSTN Tertile 1** | **MSTN Tertile 2** | **MSTN Tertile 3** | ***p*-value** |
| --- | --- | --- | --- | --- | --- |
|  | **n = 100** | **<6.46 ng/ml (n = 33)** | **6.46 – 8.69 ng/ml (n = 34)** | **>8.69 ng/ml (n = 33)** |  |
| Clinical features at presentation |  |  |  |  |  |
| Age (years) | 66.4 (56.2-77.2) | 68.0 (57.2-79.3) | 65.1 (56.0-76.5) | 65.3 (55.4-72.1) | 0.771 |
| Female | 23/100 (23.0) | 8/33 (24.2) | 7/34 (20.6) | 8/33 (24.2) | 0.919 |
| Heart rate (bpm) | 78 (70-91) | 79 (70-87) | 78 (72-93) | 78 (70-91) | 0.803 |
| Systolic blood pressure (mmHg) | 122 (106-140) | 120 (108-136) | 124 (112-143) | 124 (100-140) | 0.575 |
| eGFR (ml/min/1.73 m^2^) | 79.3 (64.4-94.2) | 87.3 (65.5-101.9) | 87.3 (77.0-96.4) | 81.4 (73.9-99.4) | 0.892 |
| Hs-cTnT >99^th^ percentile | 95/100 (95.0) | 31/33 (93.9) | 33/34 (97.1) | 31/33 (93.9) | 0.740 |
| LVEF (%) | 48.0 (40.0-60.0) | 50.0 (40.0-65.0) | 45.0 (35.0-58.8) | 46.0 (40.0-60.0) | 0.468 |
| Killip class |  |  |  |  |  |
| I | 81/98 (82.7) | 26/33 (78.8) | 30/34 (88.2) | 25/31 (80.6) | 0.492 |
| II | 10/98 (10.2) | 5/33 (15.2) | 2/34 (5.9) | 3/31 (9.7) |  |
| III | 7/98 (7.1) | 2/33 (6.1) | 2/34 (5.9) | 3/31 (9.7) |  |
| IV | 1/98 (1.0) | 0/33 (0.0) | 0/34 (0.0) | 1/31 (3.2) |  |
| Cardiometabolic risk factors |  |  |  |  |  |
| BMI (kg/m^2^) | 26.7 (24.2-29.1) | 25.7 (24.3-27.4) | 27.4 (23.9-29.9) | 26.8 (24.6-30.3) | 0.269 |
| BSA_c_ (m^2^) | 1.9 (1.8-2.0) | 1.9 (1.7-2.0) | 1.9 (1.8-2.1) | 1.9 (1.8-2.0) | 0.232 |
| Current smoker | 35/100 (35.0) | 13/33 (39.4) | 13/34 (38.2) | 9/33 (27.3) | 0.527 |
| Total cholesterol (mmol/l) | 4.6 (3.9-5.4) | 4.5 (3.9-5.2) | 4.6 (4.0-5.2) | 5.2 (3.9-5.8) | 0.338 |
| LDL-C (mmol/l) | 2.9 (2.3-3.5) | 2.8 (2.1-3.2) | 3.0 (2.4-3.5) | 3.3 (2.3-3.8) | 0.234 |
| HDL-C (mmol/l) | 1.1 (1.0-1.4) | 1.1 (0.9-1.5) | 1.1 (0.9-1.3) | 1.2 (1.1-1.3) | 0.373 |
| Triglycerides (mmol/l) | 1.0 (0.7-1.4) | 0.9 (0.8-1.2) | 0.9 (0.6-1.6) | 1.0 (0.7-1.4) | 0.910 |
| Past medical history |  |  |  |  |  |
| H_x_ of dyslipidaemia | 57/100 (57.0) | 18/33 (54.5) | 17/34 (50.0) | 22/33 (66.7) | 0.364 |
| H_x_ of hypertension | 53/100 (53.0) | 15/33 (45.5) | 16/34 (47.1) | 22/33 (66.7) | 0.107 |
| Previous PCI | 12/100 (12.0) | 3/33 (9.1) | 5/34 (14.7) | 4/33 (12.1) | 0.926 |
| Clinical chemistry and haematology |  |  |  |  |  |
| hs-CRP (mg/l) | 2.5 (1.2-9.4) | 3.3 (1.7-17.7) | 2.3 (0.9-5.9) | 2.2 (1.6-6.6) | 0.139 |
| NT-proBNP (ng/l) | 331.0 (115.8-1349.5) | 935.0 (145.0-1463.0) | 266.0 (128.5-1033.3) | 237.0 (80.0-1007.0) | 0.195 |
| hs-cTnT (ng/l) | 246.0 (71.0-705.0) | 346.0 (130.0-717.0) | 301.5 (82.3-776.0) | 118.0 (49.0-582.0) | 0.306 |
| Hb (g/dl) | 13.5 (12.8-14.3) | 13.5 (12.5-14.0) | 13.3 (12.7-14.3) | 13.7 (12.9-14.5) | 0.720 |
| Medication at presentation |  |  |  |  |  |
| Aspirin | 23/52 (44.2) | 9/19 (47.4) | 6/12 (50.0) | 8/21 (38.1) | 0.756 |
| ACEI/ARB | 27/52 (51.9) | 8/19 (42.1) | 8/12 (66.7) | 11/21 (52.4) | 0.411 |
| Betablocker | 26/52 (50.0) | 11/19 (57.9) | 7/12 (58.3) | 8/21 (38.1) | 0.368 |
| P2Y_12_ receptor inhibitor | 5/52 (9.6) | 3/19 (15.8) | 0/12 (0.0) | 2/21 (9.5) | 0.344 |
| Statin | 19/52 (36.5) | 6/19 (31.6) | 5/12 (41.7) | 8/21 (38.1) | 0.873 |

***Table S3.* Baseline characteristics of SPUM-ACS participants with acute myocardial infarction stratified by MSTN tertiles.** Data are n/N (%), or median (IQR). ACEI denotes angiotensin-converting enzyme inhibitors, ARB angiotensin receptor blockers, BMI body mass index, BSA estimated (by Dubois and Dubois) body surface area, CAD coronary artery disease, CABG coronary artery bypass grafting, CRP C-reactive protein, eGFR estimated (by CKD-EPI^45,46^) glomerular filtration rate, GDF11 growth differentiation factor 11, GRACE Global Registry of Acute Coronary Events, Hb haemoglobin, LVEF left-ventricular ejection fraction, MSTN myostatin, NT-proBNP N-terminal-pro hormone BNP, PCI percutaneous coronary intervention, and URL upper reference limit.

|  | **All Patients** | **MSTN Tertile 1** | **MSTN Tertile 2** | **MSTN Tertile 3** | ***p*-value** |
| --- | --- | --- | --- | --- | --- |
|  | **n = 100** | **<6.46 ng/ml (n = 33)** | **6.46 – 8.69 ng/ml (n = 34)** | **>8.69 ng/ml (n = 33)** |  |
| GRACE 2.0 score (%) |  |  |  |  |  |
| Death in hospital | 3.2 (1.5-5.1) | 2.9 (1.4-5.0) | 3.0 (1.5-4.7) | 3.9 (2.1-7.1) | 0.261 |
| Death at 6 months | 9.0 (4.0-15.0) | 8.0 (4.0-14.0) | 7.5 (4.0-14.0) | 9.0 (5.0-18.0) | 0.409 |
| Death at 1 year | 6.3 (3.7-12.7) | 5.7 (3.9-13.2) | 6.4 (3.2-11.0) | 6.6 (4.4-14.1) | 0.432 |
| Management delay |  |  |  |  |  |
| Onset-to-PCI (min) | 291.0 (177.0-517.3) | 375.0 (199.5-760.5) | 333.0 (181.8-483.3) | 261.0 (147.0-335.0) | 0.398 |
| Lesion characteristics |  |  |  |  |  |
| N_o_ of lesions* | 1.0 (1.0-2.0) | 1.0 (1.0-1.3) | 1.0 (1.0-2.0) | 1.0 (1.0-2.0) | 0.328 |
| N_o_ of lesions stented* | 1.0 (1.0-2.0) | 1.0 (1.0-1.0) | 1.0 (1.0-2.0) | 1.0 (1.0-2.0) | 0.154 |
| LAD occlusion | 25/100 (25.0) | 11/33 (33.3) | 6/34 (17.6) | 8/33 (24.2) | 0.405 |
| Proximal lesion | 9/100 (9.0) | 5/33 (15.2) | 1/34 (2.9) | 3/33 (9.1) | 0.906 |
| Total occlusion by main lesion^†^ | 51/90 (56.7) | 15/30 (50.0) | 17/32 (53.1) | 19/28 (67.9) | 0.344 |
| Main lesion morphology |  |  |  |  |  |
| AHA/ACC type A | 8/90 (8.9) | 1/29 (3.4) | 3/31 (9.7) | 4/30 (13.3) | 0.298 |
| AHA/ACC type B | 63/90 (70.0) | 19/29 (65.5) | 23/31 (74.2) | 21/30 (70.0) |  |
| AHA/ACC type C | 19/90 (21.1) | 9/29 (31.0) | 5/31 (16.1) | 5/30 (16.7) |  |
| Duration of hospital stay (days) | 2.0 (1.0-2.25) | 2.0 (1.0-3.0) | 2.0 (1.0-2.0) | 2.0 (1.0-2.0) | 0.895 |
| Discharge destination |  |  |  |  |  |
| Rehabilitation/other hospital | 72/99 (72.7) | 21/32 (65.6) | 24/34 (70.6) | 27/33 (81.8) | 0.377 |
| Home | 27/99 (27.3) | 11/32 (34.4) | 10/34 (29.4) | 6/33 (18.2) |  |
| Discharge medication |  |  |  |  |  |
| Aspirin | 99/99 (100.0) | 32/32 (100.0) | 34/34 (100.0) | 33/33 (100.0) | .. |
| ACEI/ARB | 92/99 (92.9) | 30/32 (93.8) | 31/34 (91.2) | 31/33 (93.9) | 0.999 |
| Betablocker | 91/99 (91.9) | 28/32 (87.5) | 31/34 (91.2) | 32/33 (97.0) | 0.388 |
| P2Y_12_ receptor inhibitor | 96/99 (97.0) | 31/32 (96.9) | 32/34 (94.1) | 33/33 (100.0) | 0.653 |
| Statin | 97/99 (98.0) | 31/32 (96.9) | 33/34 (97.1) | 33/33 (100.0) | 0.769 |
| Outcomes |  |  |  |  |  |
| MACE at 1 year‡ | 22/100 (22.0) | 11/33 (33.3) | 6/34 (17.6) | 5/33 (15.2) | 0.076 |

***Table S4.* Risk of adverse events, coronary lesion characteristics, discharge medication and 1-year MACE rates of all patients according to MSTN levels.** Data are n/N (%) or median (IQR). ACEI denotes angiotensin-converting enzyme inhibitors, ARB angiotensin receptor blockers, CABG coronary artery bypass grafting, GRACE Global Registry of Acute Coronary Events, LAD left-anterior descending coronary artery, LMWH low-molecular-weight heparin, MSTN myostatin, and PCI percutaneous coronary intervention.

*Refers to lesions with ≥75% luminal stenosis,

†Refers to first stented coronary lesion,

‡Defined as the composite of cardiac death, non-fatal myocardial infarction or ischemia-driven revascularization.

|  |  | ***B* (95% CI)** | ***p*-value*** | **SE of *B*** | **β** | **Correlations** | **Tolerance** | **VIF** | **R** | **R^2^** | **Adjusted R^2^** | ***p*-value**† | **ΔR^2^** | ***p*-value**‡ | **Durbin-Watson** |
| --- | --- | --- | --- | --- | --- | --- | --- | --- | --- | --- | --- | --- | --- | --- | --- |
| **Model 1** | Constant | 45.94 (-7.98 to 99.85) | 0.094 | 26.96 | ·· | ·· | ·· | ·· | 0.54 | 0·29 | 0·24 | <0·001^a^ | 0·29 | <0·001^a^ | ·· |
|  | Age | -0.63 (-1.35 to 0.09) | 0.083 | 0.36 | -0.20 | -0.19 | 0.94 | 1.07 |  |  |  |  |  |  |  |
|  | Sex | -7.57 (-29.52 to 14.38) | 0.49 | 10.98 | -0.08 | -0.17 | 0.89 | 1.12 |  |  |  |  |  |  |  |
|  | Hs-cTnT | 17.51 (4.74 to 30.29 | 0.008 | 6.39 | 0.31 | 0.29 | 0.94 | 1.07 |  |  |  |  |  |  |  |
|  | LAD occlusion | 30.45 (11.17 to 49.73 | 0.002 | 9.64 | 0.36 | 0.42 | 0.91 | 1.10 |  |  |  |  |  |  |  |
|  |  |  |  |  |  |  |  |  |  |  |  |  |  |  |  |
| **Model 2** | Constant | 45.86 (-8.44 to 100.16 | 0.096 | 27.15 | ·· | ·· | ·· | ·· | 0·54 | 0·29 | 0·23 | <0·001^a^ | 0·002 | 0·67^a^ | ·· |
|  | Age | -0.64 (-1.36 to 0.09 | 0.083 | 0.36 | -0.20 | -0.19 | 0.93 | 1.07 |  |  |  |  |  |  |  |
|  | Sex | -7.4 (-29.52 to 14.73 | 0.51 | 11.06 | -0.08 | -0.17 | 0.89 | 1.13 |  |  |  |  |  |  |  |
|  | Hs-cTnT | 16.3 (2.23 to 30.36 | 0.024 | 7.03 | 0.29 | 0.29 | 0.78 | 1.28 |  |  |  |  |  |  |  |
|  | LAD occlusion | 32.02 (11.27 to 52.76 | 0.003 | 10.37 | 0.38 | 0.42 | 0.80 | 1.25 |  |  |  |  |  |  |  |
|  | Onset-to-PCI | 0.01 (-0.02 to 0.03 | 0.67 | 0.01 | 0.05 | 0.01 | 0.75 | 1.33 |  |  |  |  |  |  |  |
|  |  |  |  |  |  |  |  |  |  |  |  |  |  |  |  |
| **Model 3** | Constant | 17.79 (-33.92 to 69.5 | 0.49 | 25.84 | ·· | ·· | ·· | ·· | 0·65 | 0·42 | 0·37 | <0·001^a^ | 0·13 | <0·001^a^ | 2·13 |
|  | Age | -0.92 (-1.59 to -0.24 | 0.009 | 0.34 | -0.29 | -0.19 | 0.89 | 1.13 |  |  |  |  |  |  |  |
|  | Sex | -3.84 (-24.06 to 16.38 | 0.71 | 10.11 | -0.04 | -0.17 | 0.88 | 1.14 |  |  |  |  |  |  |  |
|  | Hs-cTnT | 19.41 (6.5 to 32.32 | 0.004 | 6.45 | 0.34 | 0.29 | 0.77 | 1.30 |  |  |  |  |  |  |  |
|  | LAD occlusion | 34.26 (15.34 to 53.17 | <.001 | 9.45 | 0.40 | 0.42 | 0.80 | 1.26 |  |  |  |  |  |  |  |
|  | Onset-to-PCI | 0 (-0.02 to 0.03) | 0.74 | 0.01 | 0.04 | 0.01 | 0.75 | 1.34 |  |  |  |  |  |  |  |
|  | GDF11 | 17.55 (8 to 27.09 | <.001 | 4.77 | 0.38 | 0.25 | 0.93 | 1.08 |  |  |  |  |  |  |  |

***Table S5.* Hierarchical multiple linear regression for the prediction of myocardial infarct-size in humans.** Standardized peak CK-MB values (CK-MB x URL) served as the dependent variable. Predictors included in each model are specified in the second column, namely age (years), sex (female/male), angiographically confirmed LAD occlusion (yes/no), log-transformed hs-cTnT (ng/ml) and tertiles of GDF11. *B* denotes the unstandardized regression coefficient, β standardized coefficient, CI confidence interval, R multiple correlation coefficient, R^2^ coefficient of determination, SE standard error of *B*, and URL upper reference limit.

*Refers to each independent variable,

†Refers to each model,

‡Refers to ΔR.

^a^ANOVA F-test

|  |  | ***B* (95% CI)** | ***p*-value*** | **SE of *B*** | **β** | **Correlations** | **Tolerance** | **VIF** | **R** | **R^2^** | **Adjusted R^2^** | ***p*-value**† | **ΔR^2^** | ***p*-value**‡ | **Durbin-Watson** |
| --- | --- | --- | --- | --- | --- | --- | --- | --- | --- | --- | --- | --- | --- | --- | --- |
| **Model 1** | Constant | 45.94 (-7.98 to 99.85 | 0.094 | 26.96 | ·· | ·· | ·· | ·· | 0·54 | 0·29 | 0·24 | <0·001^a^ | 0·29 | <0·001^a^ | ·· |
|  | Age | -0.63 (-1.35 to 0.09 | 0.083 | 0.36 | -0.20 | -0.19 | 0.94 | 1.07 |  |  |  |  |  |  |  |
|  | Sex | -7.57 (-29.52 to 14.38 | 0.49 | 10.98 | -0.08 | -0.17 | 0.89 | 1.12 |  |  |  |  |  |  |  |
|  | Hs-cTnT | 17.51 (4.74 to 30.29 | 0.008 | 6.39 | 0.31 | 0.29 | 0.94 | 1.07 |  |  |  |  |  |  |  |
|  | LAD occlusion | 30.45 (11.17 to 49.73 | 0.002 | 9.64 | 0.36 | 0.42 | 0.91 | 1.10 |  |  |  |  |  |  |  |
|  |  |  |  |  |  |  |  |  |  |  |  |  |  |  |  |
| **Model 2** | Constant | 45.86 (-8.44 to 100.16 | 0.096 | 27.15 | ·· | ·· | ·· | ·· | 0·54 | 0·29 | 0·23 | <0·001^a^ | 0·002 | 0·67^a^ | ·· |
|  | Age | -0.64 (-1.36 to 0.09 | 0.083 | 0.36 | -0.20 | -0.19 | 0.93 | 1.07 |  |  |  |  |  |  |  |
|  | Sex | -7.4 (-29.52 to 14.73 | 0.51 | 11.06 | -0.08 | -0.17 | 0.89 | 1.13 |  |  |  |  |  |  |  |
|  | Hs-cTnT | 16.3 (2.23 to 30.36 | 0.024 | 7.03 | 0.29 | 0.29 | 0.78 | 1.28 |  |  |  |  |  |  |  |
|  | LAD occlusion | 32.02 (11.27 to 52.76 | 0.003 | 10.37 | 0.38 | 0.42 | 0.80 | 1.25 |  |  |  |  |  |  |  |
|  | Onset-to-PCI | 0.01 (-0.02 to 0.03 | 0.67 | 0.01 | 0.05 | 0.01 | 0.75 | 1.33 |  |  |  |  |  |  |  |
|  |  |  |  |  |  |  |  |  |  |  |  |  |  |  |  |
| **Model 3** | Constant | 34.43 (-26.86 to 95.72 | 0.27 | 30.63 | ·· | ·· | ·· | ·· | 0·55 | 0·30 | 0·23 | 0·001^a^ | 0·008 | 0·42^a^ | 2·14 |
|  | Age | -0.62 (-1.35 to 0.1 | 0.091 | 0.36 | -0.19 | -0.19 | 0.93 | 1.07 |  |  |  |  |  |  |  |
|  | Sex | -7.01 (-29.22 to 15.21 | 0.53 | 11.10 | -0.07 | -0.17 | 0.89 | 1.13 |  |  |  |  |  |  |  |
|  | Hs-cTnT | 16.29 (2.18 to 30.4 | 0.024 | 7.05 | 0.28 | 0.29 | 0.78 | 1.28 |  |  |  |  |  |  |  |
|  | LAD occlusion | 32.49 (11.64 to 53.33 | 0.003 | 10.42 | 0.38 | 0.42 | 0.80 | 1.26 |  |  |  |  |  |  |  |
|  | Onset-to-PCI | 0.01 (-0.02 to 0.03 | 0.59 | 0.01 | 0.07 | 0.01 | 0.73 | 1.36 |  |  |  |  |  |  |  |
|  | MSTN | 4.36 (-6.36 to 15.09 | 0.42 | 5.36 | 0.09 | 0.08 | 0.97 | 1.04 |  |  |  |  |  |  |  |

***Table S6.* Myocardial infarct-size, estimated by peak CK-MB, predicted by hierarchical multiple linear regression.** Standardized CK-MB (CK-MB x URL) represents the dependent variable. Predictors included in each model are specified in the second column, namely age (years), sex (female/male), angiographically confirmed LAD occlusion (yes/no), log-transformed hs-cTnT (ng/l) and tertiles of MSTN. *B* denotes the unstandardized regression coefficient, β standardized coefficient, CI confidence interval, R multiple correlation coefficient, R^2^ coefficient of determination, and SE standard error of *B*.

*Refers to each independent variable,

†Refers to each model,

‡Refers to ΔR.

^a^ANOVA F-test

**References**

1. Berlo JH van, Kanisicak O, Maillet M, Vagnozzi RJ, Karch J, Lin S-CJ, Middleton RC, Marbán E, Molkentin JD. c-kit+ cells minimally contribute cardiomyocytes to the heart. *Nature* 2014;**509**:337–341.

2. Sultana N, Zhang L, Yan J, Chen J, Cai W, Razzaque S, Jeong D, Sheng W, Bu L, Xu M, Huang G-Y, Hajjar RJ, Zhou B, Moon A, Cai C-L. Resident c-kit(+) cells in the heart are not cardiac stem cells. *Nat Commun* 2015;**6**:8701.

3. Liu Q, Yang R, Huang X, Zhang H, He L, Zhang L, Tian X, Nie Y, Hu S, Yan Y, Zhang L, Qiao Z, Wang Q-D, Lui KO, Zhou B. Genetic lineage tracing identifies in situ Kit-expressing cardiomyocytes. *Cell Res* 2016;**26**:119–130.

4. Li Y, He L, Huang X, Bhaloo SI, Zhao H, Zhang S, Pu W, Tian X, Li Y, Liu Q, Yu W, Zhang L, Liu X, Liu K, Tang J, Zhang H, Cai D, Ralf AH, Xu Q, Lui KO, Zhou B. Genetic Lineage Tracing of Nonmyocyte Population by Dual Recombinases. *Circulation* 2018;**138**:793–805.

5. Maliken BD, Molkentin JD. Undeniable Evidence That the Adult Mammalian Heart Lacks an Endogenous Regenerative Stem Cell. *Circulation* 2018;**138**:806–808.

6. Barile L, Lionetti V, Cervio E, Matteucci M, Gherghiceanu M, Popescu LM, Torre T, Siclari F, Moccetti T, Vassalli G. Extracellular vesicles fromhuman cardiac progenitor cells inhibit cardiomyocyte apoptosis and improve cardiac function aftermyocardial infarction. *Cardiovasc Res* 2014;**103**:530–541.

7. Couto G de, Gallet R, Cambier L, Jaghatspanyan E, Makkar N, Dawkins JF, Berman BP, Marbán E. Exosomal MicroRNA Transfer Into Macrophages Mediates Cellular Postconditioning. *Circulation* 2017;**136**:200–214.

8. Barile L, Cervio E, Lionetti V, Milano G, Ciullo A, Biemmi V, Bolis S, Altomare C, Matteucci M, Silvestre D Di, Brambilla F, Fertig TE, Torre T, Demertzis S, Mauri P, Moccetti T, Vassalli G. Cardioprotection by cardiac progenitor cell-secreted exosomes: role of pregnancy-associated plasma protein-A. *Cardiovasc Res* 2018;**114**:992–1005.

9. Ucar A, Gupta SK, Fiedler J, Erikci E, Kardasinski M, Batkai S, Dangwal S, Kumarswamy R, Bang C, Holzmann A, Remke J, Caprio M, Jentzsch C, Engelhardt S, Geisendorf S, Glas C, Hofmann TG, Nessling M, Richter K, Schiffer M, Carrier L, Napp LC, Bauersachs J, Chowdhury K, Thum T. The miRNA-212/132 family regulates both cardiac hypertrophy and cardiomyocyte autophagy. *Nat Commun* 2012;**3**:1078.

10. Sengupta A, Molkentin JD, Paik J-H, DePinho RA, Yutzey KE. FoxO transcription factors promote cardiomyocyte survival upon induction of oxidative stress. *J Biol Chem* 2011;**286**:7468–7478.

11. Täubel J, Hauke W, Rump S, Viereck J, Batkai S, Poetzsch J, Rode L, Weigt H, Genschel C, Lorch U, Theek C, Levin AA, Bauersachs J, Solomon SD, Thum T. Novel antisense therapy targeting microRNA-132 in patients with heart failure: results of a first-in-human Phase 1b randomized, double-blind, placebo-controlled study. *Eur Heart J* 2021;**42**:178–188.

12. Gallet R, Dawkins J, Valle J, Simsolo E, Couto G de, Middleton R, Tseliou E, Luthringer D, Kreke M, Smith RR, Marbán L, Ghaleh B, Marbán E. Exosomes secreted by cardiosphere-derived cells reduce scarring, attenuate adverse remodelling, and improve function in acute and chronic porcine myocardial infarction. *Eur Heart J* 2017;**38**:201–211.

13. Maring JA, Lodder K, Mol E, Verhage V, Wiesmeijer KC, Dingenouts CKE, Moerkamp AT, Deddens JC, Vader P, Smits AM, Sluijter JPG, Goumans MJ. Cardiac Progenitor Cell–Derived Extracellular Vesicles Reduce Infarct Size and Associate with Increased Cardiovascular Cell Proliferation. *J Cardiovasc Transl Res* Journal of Cardiovascular Translational Research; 2019;**12**:5–17.

14. Chimenti I, Smith RR, Li T-S, Gerstenblith G, Messina E, Giacomello A, Marbán E. Relative roles of direct regeneration versus paracrine effects of human cardiosphere-derived cells transplanted into infarcted mice. *Circ Res* 2010;**106**:971–980.

15. Ibrahim AG-E, Cheng K, Marbán E. Exosomes as critical agents of cardiac regeneration triggered by cell therapy. *Stem cell reports* 2014;**2**:606–619.

16. Kraler S, Wenzl FA, Georgiopoulos G, Obeid S, Liberale L, Eckardstein A von, Muller O, Mach F, Räber L, Losdat S, Schmiady MO, Stellos K, Stamatelopoulos K, Camici GG, Srdic A, Paneni F, Akhmedov A, Lüscher TF. Soluble lectin-like oxidized low-density lipoprotein receptor-1 predicts premature death in acute coronary syndromes. *Eur Heart J* 2022;**43**:1849–1860.

17. Laaksonen R, Ekroos K, Sysi-Aho M, Hilvo M, Vihervaara T, Kauhanen D, Suoniemi M, Hurme R, März W, Scharnagl H, Stojakovic T, Vlachopoulou E, Lokki ML, Nieminen MS, Klingenberg R, Matter CM, Hornemann T, Jüni P, Rodondi N, Räber L, Windecker S, Gencer B, Pedersen ER, Tell GS, Nygård O, Mach F, Sinisalo J, Lüscher TF. Plasma ceramides predict cardiovascular death in patients with stable coronary artery disease and acute coronary syndromes beyond LDL-cholesterol. *Eur Heart J* 2016;**37**:1967–1976.

18. Peng L, Gagliano-Jucá T, Pencina KM, Krishnan S, Li Z, Tracy RP, Jasuja R, Bhasin S. Age Trends in Growth and Differentiation Factor-11 and Myostatin Levels in Healthy Men, and Differential Response to Testosterone, Measured Using Liquid Chromatography–Tandem Mass Spectrometry. *Journals Gerontol Ser A* 2022;**77**:763–769.

19. Milano G, Biemmi V, Lazzarini E, Balbi C, Ciullo A, Bolis S, Ameri P, Silvestre D Di, Mauri P, Barile L, Vassalli G. Intravenous administration of cardiac progenitor cell-derived exosomes protects against doxorubicin/trastuzumab-induced cardiac toxicity. *Cardiovasc Res* 2020;**116**:383–392.

20. Balbi C, Milano G, Fertig TE, Lazzarini E, Bolis S, Taniyama Y, Sanada F, Silvestre D Di, Mauri P, Gherghiceanu M, Lüscher TF, Barile L, Vassalli G. An exosomal-carried short periostin isoform induces cardiomyocyte proliferation. *Theranostics* 2021;**11**:5634–5649.

21. Schneider CA, Rasband WS, Eliceiri KW. NIH Image to ImageJ: 25 years of image analysis. *Nat Methods* 2012;**9**:671–675.

22. Schindelin J, Arganda-Carreras I, Frise E, Kaynig V, Longair M, Pietzsch T, Preibisch S, Rueden C, Saalfeld S, Schmid B, Tinevez J-Y, White DJ, Hartenstein V, Eliceiri K, Tomancak P, Cardona A. Fiji: an open-source platform for biological-image analysis. *Nat Methods* 2012;**9**:676–682.

23. Krämer A, Green J, Pollard J, Tugendreich S. Causal analysis approaches in Ingenuity Pathway Analysis. *Bioinformatics* 2014;**30**:523–530.

24. Krämer A, Green J, Billaud J-N, Pasare NA, Jones M, Tugendreich S. Mining hidden knowledge: embedding models of cause–effect relationships curated from the biomedical literature. *Bioinforma Adv* 2022;**2**:vbac022.

25. Loffredo FS, Steinhauser ML, Jay SM, Gannon J, Pancoast JR, Yalamanchi P, Sinha M, Dall’Osso C, Khong D, Shadrach JL, Miller CM, Singer BS, Stewart A, Psychogios N, Gerszten RE, Hartigan AJ, Kim MJ, Serwold T, Wagers AJ, Lee RT. Growth differentiation factor 11 is a circulating factor that reverses age-related cardiac hypertrophy. *Cell* 2013;**153**:828–839.

26. Bujak M, Ren G, Kweon HJ, Dobaczewski M, Reddy A, Taffet G, Wang X-F, Frangogiannis NG. Essential role of Smad3 in infarct healing and in the pathogenesis of cardiac remodeling. *Circulation* 2007;**116**:2127–2138.

27. Song L, Yan W, Chen X, Deng C, Wang Q, Jiao K. Myocardial smad4 is essential for cardiogenesis in mouse embryos. *Circ Res* 2007;**101**:277–285.

28. Morimoto H, Takahashi M, Shiba Y, Izawa A, Ise H, Hongo M, Hatake K, Motoyoshi K, Ikeda U. Bone marrow-derived CXCR4+ cells mobilized by macrophage colony-stimulating factor participate in the reduction of infarct area and improvement of cardiac remodeling after myocardial infarction in mice. *Am J Pathol* 2007;**171**:755–766.

29. Schmittgen TD, Livak KJ. Analyzing real-time PCR data by the comparative CT method. *Nat Protoc* 2008;**3**:1101–1108.

30. Benjamini Y, Hochberg Y. Controlling the False Discovery Rate: A Practical and Powerful Approach to Multiple Testing. *J R Stat Soc Ser B* [Royal Statistical Society, Wiley]; 1995;**57**:289–300.

31. Claycomb WC, Lanson NA, Stallworth BS, Egeland DB, Delcarpio JB, Bahinski A, Izzo NJ. HL-1 cells: a cardiac muscle cell line that contracts and retains phenotypic characteristics of the adult cardiomyocyte. *Proc Natl Acad Sci U S A* 1998;**95**:2979–2984.

32. Smith SC, Zhang X, Zhang X, Gross P, Starosta T, Mohsin S, Franti M, Gupta P, Hayes D, Myzithras M, Kahn J, Tanner J, Weldon SM, Khalil A, Guo X, Sabri A, Chen X, MacDonnell S, Houser SR. GDF11 does not rescue aging-related pathological hypertrophy. *Circ Res* 2015;**117**:926–932.

33. Zhao L, Zhang S, Cui J, Huang W, Wang J, Su F, Chen N, Gong Q. TERT assists GDF11 to rejuvenate senescent VEGFR2+/CD133+ cells in elderly patients with myocardial infarction. *Lab Invest* 2019;**99**:1661–1688.

34. Mahendiran T, Klingenberg R, Nanchen D, Gencer B, Meier D, Räber L, Carballo D, Matter CM, Lüscher TF, Mach F, Rodondi N, Muller O, Fournier S. CCN family member 1 (CCN1) is an early marker of infarct size and left ventricular dysfunction in STEMI patients. *Atherosclerosis* 2021;**335**:77–83.

35. Thygesen K, Alpert JS, White HD, Joint ESC/ACCF/AHA/WHF Task Force for the Redefinition of Myocardial Infarction, Jaffe AS, Apple FS, Galvani M, Katus HA, Newby LK, Ravkilde J, Chaitman B, Clemmensen PM, Dellborg M, Hod H, Porela P, Underwood R, Bax JJ, Beller GA, Bonow R, Wall EE Van der, Bassand J-P, Wijns W, Ferguson TB, Steg PG, Uretsky BF, Williams DO, Armstrong PW, Antman EM, Fox KMKA, Hamm CW, et al. Universal definition of myocardial infarction. *Circulation* 2007;**116**:2634–2653.

36. Elm E von, Altman DG, Egger M, Pocock SJ, Gøtzsche PC, Vandenbroucke JP, STROBE Initiative. The Strengthening the Reporting of Observational Studies in Epidemiology (STROBE) statement: guidelines for reporting observational studies. *J Clin Epidemiol* 2008;**61**:344–349.

37. Vaccarino V, Parsons L, Every NR, Barron H V, Krumholz HM. Sex-based differences in early mortality after myocardial infarction. National Registry of Myocardial Infarction 2 Participants. *N Engl J Med* 1999;**341**:217–225.

38. Stone GW, Dixon SR, Grines CL, Cox DA, Webb JG, Brodie BR, Griffin JJ, Martin JL, Fahy M, Mehran R, Miller TD, Gibbons RJ, O’Neill WW. Predictors of infarct size after primary coronary angioplasty in acute myocardial infarction from pooled analysis from four contemporary trials. *Am J Cardiol* 2007;**100**:1370–1375.

39. Panteghini M, Cuccia C, Bonetti G, Giubbini R, Pagani F, Bonini E. Single-point cardiac troponin T at coronary care unit discharge after myocardial infarction correlates with infarct size and ejection fraction. *Clin Chem* 2002;**48**:1432–1436.

40. Grande P, Hansen BF, Christiansen C, Naestoft J. Estimation of acute myocardial infarct size in man by serum CK-MB measurements. *Circulation* 1982;**65**:756–764.

41. Dohi T, Maehara A, Brener SJ, Généreux P, Gershlick AH, Mehran R, Gibson CM, Mintz GS, Stone GW. Utility of peak creatine kinase-MB measurements in predicting myocardial infarct size, left ventricular dysfunction, and outcome after first anterior wall acute myocardial infarction (from the INFUSE-AMI trial). *Am J Cardiol* 2015;**115**:563–570.

42. Hair JF, Black WC, Babin BJ, Anderson RE. Multivariate data analysis. 7th editio. Harlow, England: Pearson; 2014.

43. Huber PJ. Robust statistics. New York: John Wiley & Sons; 1981.

44. Cook RD, Weisberg S. Residuals and influence in regression. New York: Chapman & Hall;

45. Levey AS, Stevens LA, Schmid CH, Zhang YL, Castro AF, Feldman HI, Kusek JW, Eggers P, Lente F Van, Greene T, Coresh J, CKD-EPI (Chronic Kidney Disease Epidemiology Collaboration). A new equation to estimate glomerular filtration rate. *Ann Intern Med* 2009;**150**:604–612.

46. Inker LA, Schmid CH, Tighiouart H, Eckfeldt JH, Feldman HI, Greene T, Kusek JW, Manzi J, Lente F Van, Zhang YL, Coresh J, Levey AS, CKD-EPI Investigators. Estimating glomerular filtration rate from serum creatinine and cystatin C. *N Engl J Med* 2012;**367**:20–29.
